# An Evidence-Tiered Developmental Framework for Congenital Vertebral Malformations: Spatiotemporal Thresholds in Genotype–Phenotype Interpretation

**DOI:** 10.3390/genes17101309

**Published:** 2026-10-21

**Authors:** Xuhao Wang, Qi Zhang, Enze Ji, Yuchao Sui, Qingshuo Zhu, Kai Li, Xuhong Xue

**Affiliations:** Department of Orthopedics, The Second Hospital of Shanxi Medical University, Taiyuan 030001, China

**Keywords:** congenital vertebral malformation, developmental anomaly, genotype–phenotype correlation, somitogenesis, developmental threshold, *TBX6*, variant interpretation

## Abstract

Congenital vertebral malformations (CVMs) encompass formation, segmentation and mixed defects, with or without scoliosis. This narrative review relates human genetic findings to paraxial mesoderm specification, somitogenesis, sclerotome differentiation and vertebral morphogenesis. An author-defined evidence hierarchy distinguishes established disease relationships, emerging susceptibility mechanisms and hypothesis-generating observations; it is not a formal ClinGen classification. Replicated *TBX6* dosage mechanisms and disease-specific segmentation disorders provide stronger etiological evidence than candidate-gene burden signals, regulatory hypotheses or accessible-tissue methylation associations. Evidence is assigned to a defined gene–disease or mechanism–phenotype relationship, not to a gene or pathway universally. Building on previous developmental and gene–environment models, we outline a conceptual spatiotemporal threshold framework in which dosage, developmental stage, tissue distribution and modifiers may influence vertebral anatomy. No numerical human threshold has been established. Explicit appraisal criteria, relationship-specific evidence summaries and proposed experimental tests separate demonstrated findings from extrapolation. Standardized vertebral and rib phenotyping, associated anomalies and inheritance remain essential for genomic interpretation. Molecular diagnosis may inform counseling and syndrome-specific evaluation but does not independently establish prognosis or surgical indications.

## 1. Introduction

Congenital vertebral malformations (CVMs) are structural vertebral abnormalities arising from abnormal formation, segmentation or both during development [1,2]. For this review, CVM denotes the documented vertebral defect regardless of whether a coronal spinal curve is present. Structural morphology is classified as a formation, segmentation or mixed defect. Clinical context is recorded separately as isolated or non-isolated, with a named syndrome specified when established; each morphological category can occur in either context. Hemivertebrae, wedge and butterfly vertebrae, block vertebrae and unilateral unsegmented bars illustrate this spectrum [1,3]. Congenital scoliosis denotes a deformity associated with a congenital structural cause; it is not a synonym for every CVM. Neural, genitourinary and cardiovascular findings reported in referral or surgical series cannot be generalized to all people with CVMs [4,5].

**Figure 1:**
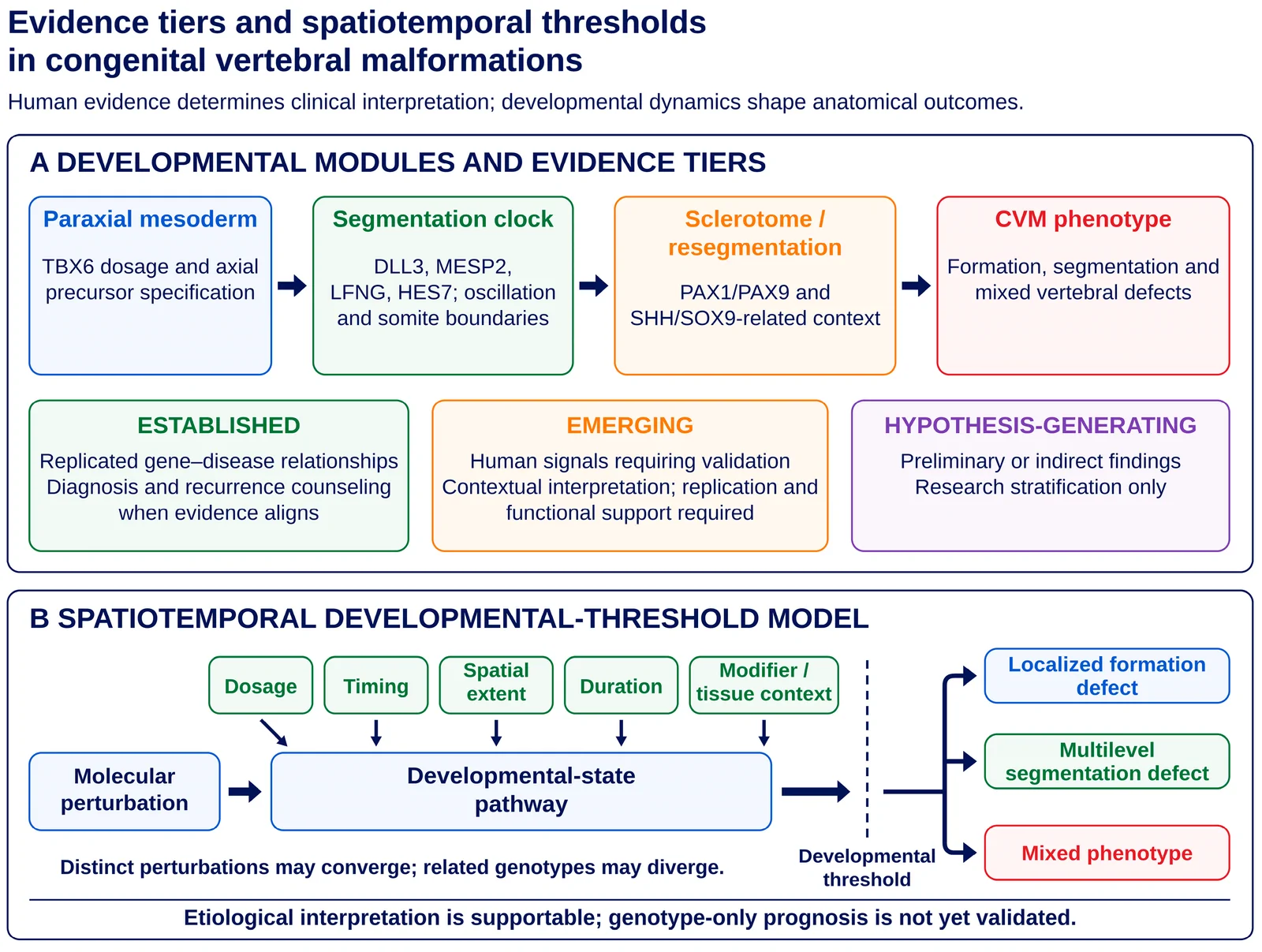
Evidence tiers and spatiotemporal developmental thresholds in congenital vertebral malformations. (**A**) Developmental modules and the established, emerging and hypothesis-generating evidence categories are summarized. Tier assignments apply to specified gene–disease or genotype–phenotype relationships, including inheritance and variant mechanism; operational criteria and evidence appraisals are provided in Table 1, Tables S2 and S5. (**B**) The proposed threshold model places molecular perturbation in the context of dosage, timing, spatial extent, duration and modifier or tissue context. Distinct perturbations may converge on similar phenotypes, whereas related genotypes may diverge. The threshold is a conceptual hypothesis, not a measured human cutoff or validated clinical tool. The framework informs etiological interpretation but does not establish genotype-only prediction of progression, surgical need or long-term function.

Figure 1 summarizes the developmental sequence, evidence categories and proposed spatiotemporal threshold framework. The diagram provides a conceptual overview; the criteria and appraisals for specific human disease relationships are given in Table 1, Tables S2 and S5.

**Figure 2:**
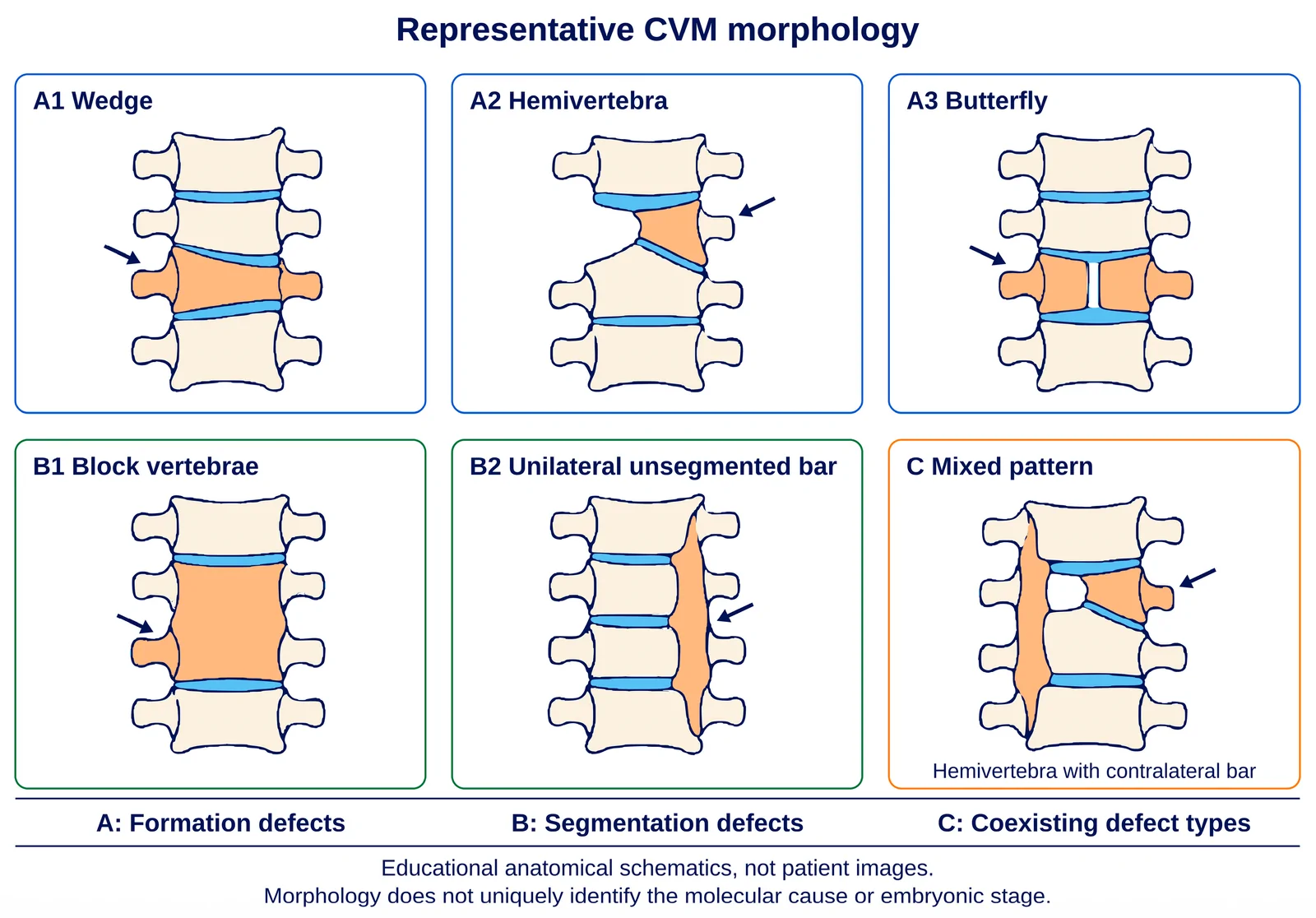
Representative vertebral body patterns illustrate formation, segmentation and mixed CVMs. (**A1**–**A3**) show wedge, hemivertebra and butterfly vertebra; (**B1**,**B2**) show block vertebrae and a unilateral unsegmented bar; (**C**) combines a hemivertebra with a contralateral bar. They are not patient images or diagnostic radiographs and do not imply a unique molecular cause. Arrows and orange shading indicate the illustrated vertebral abnormalities.

Clinical classification relies on radiographic morphology [6]. Figure 2 illustrates representative formation, segmentation and mixed patterns as educational schematics, not patient radiographs. Anatomy remains essential for surveillance and surgical planning but does not identify the embryonic event or molecular cause. Similar morphology may arise from distinct processes, and one disease relationship may have variable expression. Morphological classification is therefore the starting point for etiological interpretation rather than a molecular diagnosis.

Human genetic evidence is strongest when considered within a specific disease and inheritance model. *TBX6* compound inheritance links a rare disruptive allele and a hypomorphic background to congenital scoliosis [7,8,9,10]. Autosomal recessive spondylocostal dysostosis (SCDO) includes *DLL3*-related SCDO1 (MIM 277300), *MESP2*-related SCDO2 (MIM 608681), *LFNG*-related SCDO3 (MIM 609813) and *HES7*-related SCDO4 (MIM 613686) [11,12,13]. Their shared clinical core comprises multiple vertebral segmentation abnormalities and abnormal ribs, often with a short trunk; *LFNG*-related disease can have severe axial involvement, whereas gene-specific and allelic differences preclude a single invariant pattern [12,14,15,16]. These are phenotype MIM numbers, not gene identifiers. Table 1 separates these relationships from candidate associations and experimental developmental roles.

The four established SCDO relationships have overlapping but not identical radiographic descriptions. *DLL3*-related SCDO commonly shows generalized irregular vertebral segmentation with rib abnormalities. In *MESP2*-related SCDO, all vertebral segments show at least some disruption, but lumbar vertebrae are relatively mildly affected compared with thoracic vertebrae, and in the limited reports the ribs tend to be straight and more regularly aligned, with fewer points of fusion. *LFNG*-related disease has been reported with marked spinal shortening and severe segmentation throughout the spine, whereas the limited *HES7* series ranges from SCDO-like to spondylothoracic-like patterns with widespread segmentation defects [11]. Allelic *MESP2*-related spondylothoracic dysostosis (STD) is described as a distinct phenotype, with more severe shortening of the whole spine, especially the thoracic spine, rib fusions at the posterior costovertebral origins and ribs that fan out in a “crab-like” configuration; most reported individuals carry biallelic nonsense variants in *MESP2* exon 1 [11]. These are reported tendencies, not gene-specific imaging tests or guarantees of severity in an individual.

The developmental sequence relevant to CVMs extends beyond somite segmentation. Somites must acquire rostrocaudal identity, differentiate into sclerotome, resegment, condense and form vertebral and intervertebral disk structures under coordinated signaling and transcriptional control [17,18]. *PAX1*/*PAX9*, SHH, *SOX9*, *HOX* programs, extracellular matrix regulation and tissue polarity thus add further mechanistic layers. Some of these components underlie established human disorders; others remain candidates supported mainly by experimental biology or association studies.

Recent reviews describe the molecular landscape, genetic and epigenetic associations, and classification of congenital spinal deformity [19,20,21,22]. Threshold reasoning itself has experimental antecedents, including gene–environment interaction and planar cell polarity models [23,24]. The present emphasis is an operational appraisal of particular disease relationships, explicit separation of variant classification from relationship validity, and testable boundaries for clinical interpretation. Table S4 compares this emphasis with prior reviews.

**Figure 3:**
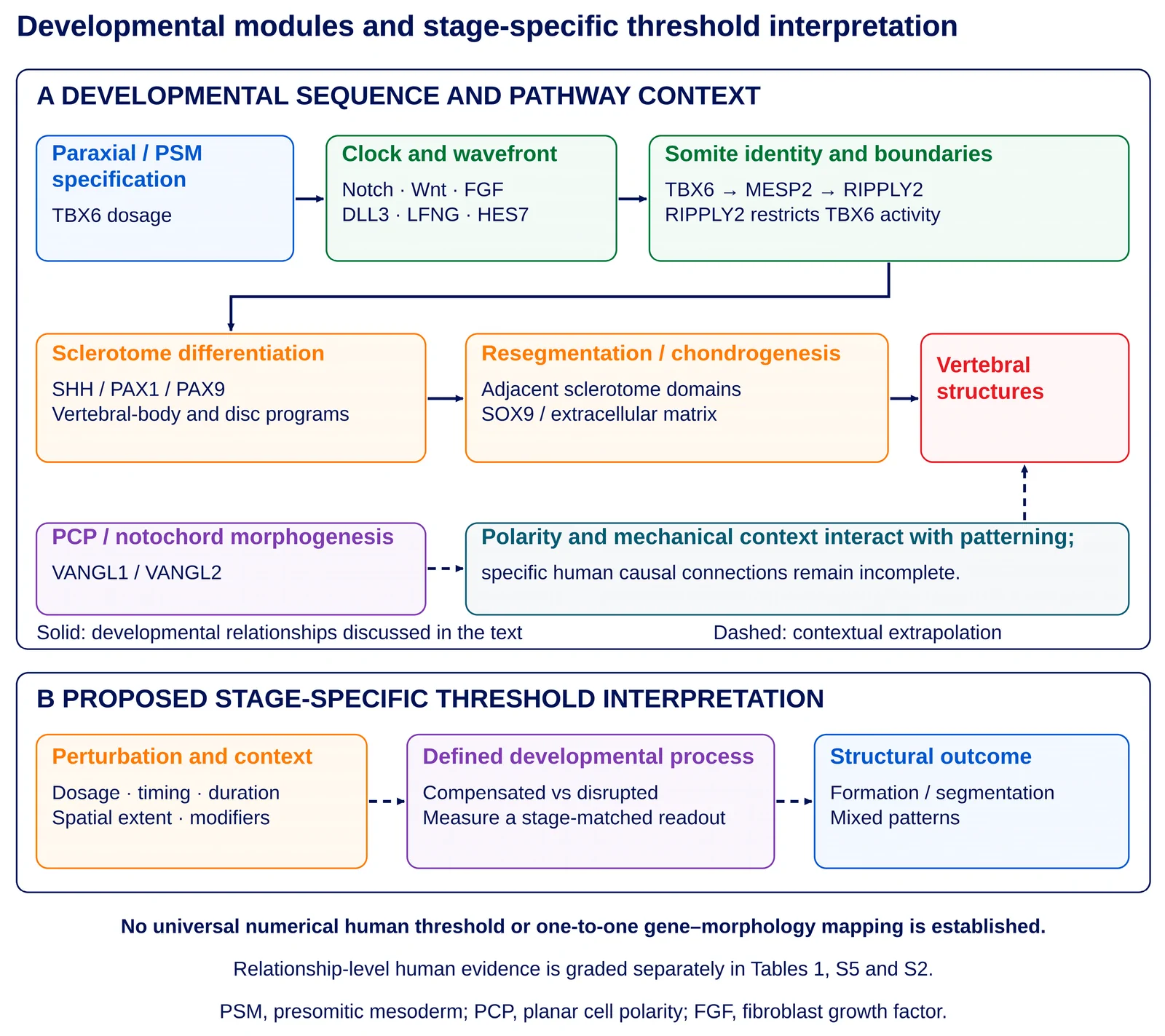
Developmental modules provide a biological context for evidence appraisal, not a gene-wide diagnostic grade. (**A**) The diagram locates segmentation-clock signaling, *TBX6*–*MESP2*–*RIPPLY2* boundary regulation, sclerotome differentiation and tissue polarity within the developmental sequence. Solid connections summarize developmental relationships discussed in Section 4; dashed connections identify contextual extrapolation. (**B**) Dosage, timing, duration and spatial extent are proposed modifiers of a stage-specific developmental response. The threshold is conceptual, not a measured human cutoff; disease-specific human evidence is summarized separately in Table 1.

**Figure 4:**
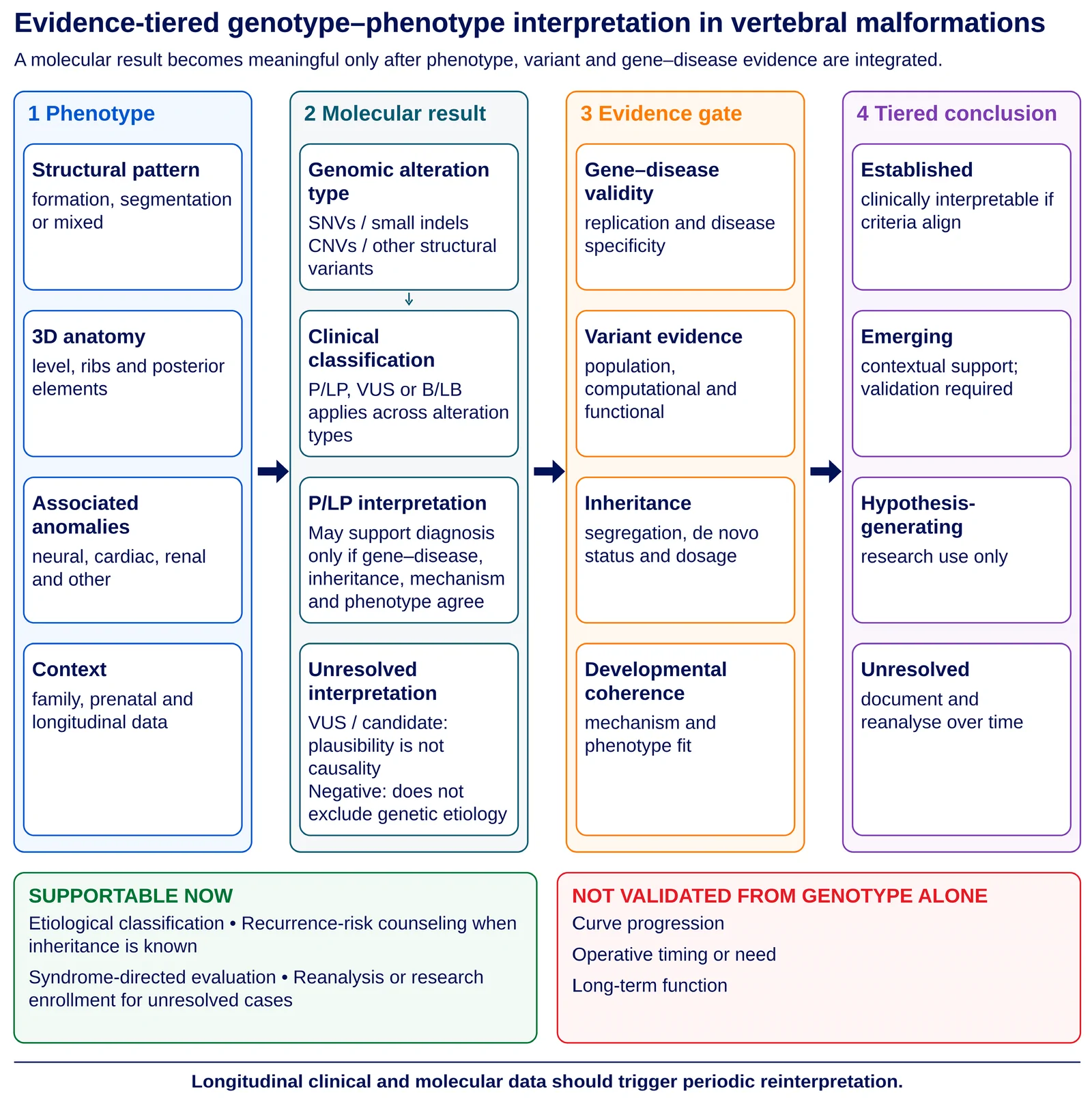
Evidence-tiered genotype–phenotype interpretation in congenital vertebral malformations. The molecular-result panel distinguishes genomic alteration type from clinical classification, using the appropriate sequence-variant or CNV standards [26,27]. A P/LP finding may support a molecular diagnosis only when the gene–disease relationship, inheritance, variant mechanism and patient phenotype are concordant. VUS and candidate findings do not independently establish a diagnosis; a negative test does not exclude a genetic etiology. The relationship-specific evidence tiers do not replace variant classification; “unresolved” denotes an interpretation status, not a fourth evidence tier. The original phenotype, evidence-assessment and clinical-use framework is retained, including the distinction between etiological interpretation and unvalidated genotype-only prognosis. SNV, single-nucleotide variant; indel, insertion/deletion; CNV, copy-number variant; P/LP, pathogenic/likely pathogenic; VUS, variant of uncertain significance; B/LB, benign/likely benign.

The review links two questions: how well a particular molecular relationship is supported in humans and how developmental context might explain variable anatomy. Established findings, mechanistic extrapolations and new predictions are kept separate. Table S5 defines the appraisal rules; Table 1 applies them to 26 named relationships; Table S2 additionally records three broad exploratory observation categories, which are not graded as individual gene–disease relationships. Figure 1 provides an overview of the evidence-tiered developmental framework. Figure 3 maps the developmental modules and the conceptual threshold interpretation, while Figure 4 separates genomic alteration type, clinical classification and phenotype concordance.

## 2. Literature Search and Evidence Framework

This is a selective narrative review. Original literature identification, relevance screening and initial narrative evidence appraisal were conducted by Xuhao Wang alone, without independent duplicate screening. PubMed/MEDLINE was the primary database; Web of Science, Embase, Scopus and Google Scholar were used for supplementary searches. The final search date was 31 May 2026 for all sources, and no language restrictions were applied. Eight PubMed/MEDLINE search strategies were recovered from retained author records and are reproduced in Supplementary Table S1c. These strategies represent only part of the original search history. Other original PubMed/MEDLINE queries, the search terms used in the supplementary databases, and the numbers of records identified, deduplicated, screened, excluded and included were not retained. Table S1a–c document the available original information and the separate revision-stage retrieval. The original selection process cannot be fully reconstructed, and the completeness of retrieval cannot be established.

Revision-stage source checking used PubMed-indexed records retrieved through the Europe PMC API on 12 October 2026. Eleven topic-specific queries applied the SRC:MED restriction, a first-publication-date interval from 1 January 1900 to 12 October 2026, and no language filter. Table S1b provides the exact queries, limits, hit counts and retrieval counts. Deduplication by indexed record identifier yielded 2207 unique records. These form a source-verification pool, not a count of included or systematically screened studies. A broad vertebral–cardiac query returned 1596 hits, of which 1000 were retrieved because of the cap; a narrower CVM-focused cardiac query retrieved all 90 returned records. The focused query does not establish complete retrieval of the broad query.

Human genetic reports were eligible for relationship appraisal when they documented a congenital structural vertebral phenotype and supplied interpretable genetic or inheritance information. Morphology (formation, segmentation or mixed) and clinical context (isolated or non-isolated, with any established syndrome recorded separately) were considered as separate descriptors when the source allowed this. Congenital scoliosis cohorts without a documented structural vertebral defect were used only as clinical or methodological context, not as proof of a CVM gene–disease relationship. Studies confined to idiopathic or acquired scoliosis were excluded from that appraisal. Experimental studies, clinical imaging studies, reviews, disease summaries and guidance informed mechanism or interpretation but were not counted as independent human genetic replication. Re-reported or potentially overlapping families and cohorts were not counted twice.

Source verification focused on the original references and selected primary reports relevant to specific manuscript claims. Indexed metadata and abstracts were checked, and full texts were examined when accessible for numerical, segregation and functional details. Unverified details were omitted or kept at a directional level. An exhaustive eligibility screen of the 2207-record pool was not documented, and historical included/excluded totals are unavailable. No formal risk-of-bias assessment, quantitative evidence score or pooled diagnostic yield was applied. The documented retrieval improves traceability, while the review retains its narrative and single-author limitations. A separate report-level pedigree check was completed during revision on 14 October 2026 for the eight Table 1b relationships with previously unreported counts; its scope, retained units, overlaps and allele-level limits are specified in Table S7.

Evidence tiers apply to a specified human gene–disease or genotype–phenotype relationship, including its inheritance and variant mechanism, rather than universally to a gene, pathway or allele. Established requires all four conditions: E1, direct human documentation of the target congenital structural vertebral phenotype; E2, at least one independently ascertained, non-overlapping human family or cohort beyond discovery supporting the same relationship; E3, coherent genetic support for the specified inheritance and mechanism; and E4, no unresolved credible contradiction that materially undermines that relationship. Table S5 specifies how each condition is evaluated, and Table S2b records its application and limitations for every entry. Two independent data sources are a minimum replication condition, not sufficient proof on their own; no universal family- or case-number cutoff is imposed. In practice, each Mendelian relationship classified as Established in this review is supported by five to 14 non-duplicate pedigrees in the audited reports (Table S7; syndromic pedigrees for *PAX1* and *NADSYN1*), and the *TBX6* compound-inheritance relationship is supported by several independently ascertained case series. Within-series recurrence and duplicate reporting are not independent replication.

Emerging denotes direct human genetic support for a plausible specified relationship, while at least one established condition remains unmet or incompletely resolved; the unresolved condition is recorded in Table S2b. The minimum for Emerging is E1 met with human germline genetic evidence plus at least one of the following: a statistically significant controlled case–control association; co-segregation of the alteration with the phenotype in at least one family; or functional evidence specific to the patient alteration, such as an assay of the patient allele or a model reproducing the same dosage change. Hypothesis-generating applies when this minimum is not met, with interpretation resting mainly on exploratory, experimental or indirect observations. Non-genetic molecular associations, such as DNA methylation measured in accessible postnatal tissue, cannot exceed this tier because they establish neither an inherited cause nor the direction of causation. An assignment may be withheld when a proposed relationship is materially contradicted or cannot be meaningfully evaluated, rather than forced into a positive tier. Table S5 details evaluation of phase, segregation, penetrance, expressivity, functional model and allele specificity, and conflicting evidence; Table S2 records 26 named relationship assessments and three broad exploratory observation categories, each with its evidence boundary; an exploratory category is not a validity claim for every locus or allele within it. Functional evidence informs mechanisms but cannot replace human replication, while the lack of a rescue experiment alone does not negate strong human genetic evidence.

Relationships are grouped only when the gene or locus, inheritance, target disease spectrum and proposed variant mechanism are compatible. Different alleles can support the same established impaired-function relationship; recurrence of the identical allele is not universally required. Conversely, biallelic missense combinations are not assumed to share one validated mechanism merely because they involve the same gene. This distinction explains why the noncanonical *TBX6* combinations remain separate from the canonical compound-inheritance model and why mechanism-level uncertainty is retained within the noncanonical row. A named syndromic relationship includes reported vertebral involvement but does not require every affected family to have a vertebral defect or establish isolated-CVM causality.

ClinGen provides a formal framework for gene–disease validity, whereas ACMG/AMP sequence and ACMG/ClinGen CNV standards classify individual variants [25,26,27]. The three tiers used here are an author-defined qualitative appraisal, not equivalent to ClinGen categories or a substitute for variant classification. Clinical utility, including diagnostic interpretation, counseling, prognosis and management, requires separate evidence. Xuhao Wang made the initial tier assignments. During revision, Qi Zhang independently reassessed all 29 Table S2 appraisal entries (26 named relationships and three exploratory categories) against the criteria in use for that reassessment. The two authors agreed on all 29 tier labels; no tier disagreement required adjudication. The original literature search and relevance screening were not independently duplicated.

Use of generative artificial intelligence tools. OpenAI ChatGPT/Codex assisted language revision and organization. No new patient measurements or experimental results were generated. Outputs were treated as draft material, with scientific support drawn from cited publications rather than generated text. Revision-stage assistance occurred in October 2026; exact model and application-build identifiers for the original preparation sessions were not retained. The authors retain responsibility for scientific interpretation and final verification of the manuscript and figures.

## 3. Genetic Architecture and Strength of Human Evidence

### 3.1. Paraxial Mesoderm Specification and the Clock–Boundary Pathway

Table 1 organizes human evidence by developmental function while grading each specified relationship separately. In the paraxial mesoderm module, *TBX6* provides a replicated compound-inheritance model: a rare loss-of-function allele, including deletion, can act with a common hypomorphic allele to increase susceptibility [7,8,10]. This relationship is not interchangeable with biallelic loss of function, isolated heterozygous variation or dosage gain [28,29]. The *TBX6*-containing 16p11.2 locus illustrates how a recurrent structural variant and the allele on the other chromosome must be interpreted together [8,30].

*TBX6*-associated congenital scoliosis (TACS) has been described as a recognizable imaging subgroup, and TACScore has been evaluated for prioritizing genetic testing [31,32]. Evidence for the compound-inheritance relationship includes studies in Chinese and Japanese populations, but cohort location is not a substitute for measured genetic ancestry [8,9,10]. Hypomorphic haplotype frequencies, linkage disequilibrium and modifier backgrounds may differ across populations. Population-specific allele frequencies and diverse-ancestry replication are needed before transporting risk estimates or counseling assumptions. Preliminary surgical outcome analyses are not equivalent to externally validated prognostic performance [33].

Within the clock–boundary module, Notch-related *DLL3*, *LFNG* and *HES7* disease relationships and *MESP2*-dependent somite patterning explain distinct recessive segmentation syndromes [12,13,14,15,16,34]. *RIPPLY2* is a boundary regulator linked to biallelic cervical and cervicothoracic malformations, including posterior cervical abnormalities and variable rib involvement [35,36,37]. The table does not group these genes under one grade. In particular, a supported *RIPPLY2* disease relationship does not resolve every allele: the 2021 report classified the recurrent splice-region variant as uncertain [37]. Gene–disease validity cannot substitute for variant-level classification.

*DLL1* also participates in Notch signaling, but the replicated human disorder described with heterozygous pathogenic variants is primarily neurodevelopmental, with variable multisystem features [38]. The report includes a radiographically documented congenital lumbar segmentation defect and hemivertebra in one syndromic individual; its broader scoliosis findings should not all be assumed to be CVMs. *DLL1* is therefore not given an established isolated-CVM designation solely because it belongs to the same pathway. A separate report described two brothers who were homozygous for *DLL1* p.Gly512Arg with congenital vertebral segmentation defects [39]. Because this recessive relationship currently rests on one family and mechanistic readouts from patient samples, we grade it as Emerging rather than extending the heterozygous neurodevelopmental evidence to it. In contrast, a characteristic multilevel vertebral–rib phenotype can support focused interpretation of the recessive SCDO relationships, subject to the appropriate allele and inheritance evidence [11].

Established relationship evidence supports etiological interpretation only when the patient phenotype, inheritance, variant mechanism and allele classification are concordant. Functional pathway membership alone is insufficient. Table S2 records the human evidence, segregation, experimental support and principal limitations separately, so a strong experimental developmental role cannot conceal weak human association.

### 3.2. Complex Inheritance, Structural Variation and Emerging Susceptibility Genes

Large sequencing studies indicate that CVMs are highly locus-heterogeneous and that many cases remain genetically unresolved [19,40,41]. The etiological spectrum spans Mendelian disorders, dosage-sensitive loci, rare-variant burden, possible oligogenic effects and regulatory variation. A negative targeted panel therefore does not exclude a genetic contribution, particularly when the phenotype is complex or multisystem.

Structural variation must be interpreted at a defined locus rather than assigned a class-wide evidence tier. The recurrent 16p11.2 interval includes *TBX6* and has direct evidence relevant to compound-inheritance CVM [8]. Other CNV findings in congenital scoliosis cohorts may identify candidates rather than establish each region as causal [42]. Syndromic chromosomal findings, including 22q11.2 deletion, require whole-phenotype interpretation rather than automatic attribution of every vertebral finding [43]. The named SCDO loci are *DLL3* at 19q13.2, *MESP2* at 15q26.1, *LFNG* at 7p22.3, *HES7* at 17p13.1, *RIPPLY2* at 6q14.2 and *TBX6* at 16p11.2 [11]. These positions identify disease genes, not independent evidence that an entire chromosomal band causes CVM. CNV classification uses dedicated ACMG/ClinGen standards; non-coding sequence variation requires different evidence and is discussed separately [27].

Burden analyses have nominated *ITPR2*, *TPO*, *H6PD* and *SEC24B* as CVM risk candidates [40]. Other reports address *NADSYN1*, *DSTYK*, *COL11A2*, *FBN1* and *SOX9* in different disease or experimental contexts [44,45,46,47,48]. These findings should not be collapsed into equivalent CVM diagnoses. For example, established syndromic relationships of a gene do not automatically validate an isolated-CVM burden signal or a particular missense allele. Table S2 distinguishes human association, syndrome-specific evidence and model-system observations, with replication and variant-specific limitations retained.

*VANGL1* and *VANGL2* belong to the tissue-polarity module but are listed separately in Table 1. Human rare-variant findings and developmental experiments support an emerging susceptibility interpretation, not a general fully penetrant monogenic CVM model [23]. The experiments include a selected *Vangl1* susceptibility allele and *Vangl2* dosage perturbations; neither model validates all alleles in either gene. Limited parental information and incompletely defined penetrance remain important constraints.

Non-coding and oligogenic models remain especially difficult to evaluate. A regulatory variant may alter expression only during a narrow developmental interval, and several modest perturbations may converge before any single variant reaches conventional pathogenicity criteria. A *TBX6*-centered mutational-burden study provides one example of an oligogenic hypothesis, but broader validation is still required [49]. Computational annotation can prioritize enhancers, transcription-factor motifs and dosage-sensitive regions, but prioritized non-coding variants still require disease-relevant functional follow-up before causal interpretation [50]. Family-based sequencing, allele-specific expression and perturbation of candidate regulatory elements in developmentally relevant models will be needed to establish whether these variants are causal.

### 3.3. Sclerotome Differentiation and Regulatory Evidence

The sclerotome module illustrates why evidence must be disease-specific. Experimental *Pax1* deficiency and combined *Pax1*/*Pax9* loss affect vertebral and intervertebral disk development [51,52,53,54]. Human biallelic *PAX1* variants are an established cause of otofaciocervical syndrome, in which vertebral findings occur within a broader craniofacial, hearing and other systemic phenotype [55,56]. This supports syndrome-specific interpretation, not a universal isolated-CVM designation. *PAX9* remains a developmental candidate for CVM in this appraisal. A deletion of *Pax1* regulatory elements at a scoliosis-associated locus produced female-biased caudal abnormalities in mice; that finding is not equivalent to a generalized human congenital vertebral syndrome [57].

Epigenetic reports in selected congenital scoliosis populations include *TNS3* and *KAT6B* methylation associations and genome-wide methylation differences [58,59,60]. These observations do not establish epigenetic causation across all CVMs. Methylation may be a primary contributor, a modifier or a consequence of altered tissue state, treatment or cell composition. Small samples, heterogeneous accessible tissues and the lack of embryonic axial measurements prevent these alternatives from being distinguished. These relationships are hypothesis-generating, not diagnostic or prognostic biomarkers.

The same separation applies to regulatory and oligogenic proposals: developmental plausibility supports prioritization, whereas independent human association, segregation and variant-specific functional evidence determine causal confidence. Table 1 summarizes the principal relationships, and Table S2 provides the supporting appraisal and limitations. Experimental pathway importance and clinical disease validity are deliberately not treated as synonyms.

**Table 1:** (**a**). Relationship-specific genetic, functional and tier evidence relevant to congenital vertebral malformations. (**b**) Human replication, segregation and clinical interpretation for the relationships in Table 1a.

(**a**)				
Gene/Locus	Specified Phenotype/Inheritance	Human Evidence/Replication	Functional or Developmental Support	Tier/Principal Limitation
*TBX6*	Rare loss of function/deletion plus T-C-A in trans; CVM/CS	Independent human series support the complete compound genotype [7,8,10].	Dosage and reporter studies support reduced TBX6 activity.	Established; applies to the complete genotype, not either component alone.
*TBX6*	Noncanonical biallelic missense combinations; vertebral/rib defects	Several cases and recurrent alleles; exact combination-level replication remains limited [28,61,62].	Transcription, localization and patient-cell findings vary by allele.	Emerging; a shared validated mechanism cannot be assumed across different missense combinations.
*16p11.2 dup*	Copy-number gain including *TBX6*; cervical CVM susceptibility	Four of seven selected carriers had CVMs involving the cervical spine [29]. Earlier duplication reports described spinal phenotypes, but their comparability with this specific cervical pattern remains uncertain.	*Tbx6* overexpression models support dosage plausibility.	Emerging; multigene CNV prevents *TBX6*-specific attribution or a penetrance estimate.
*DLL3*	Biallelic variants; SCDO1 with vertebral/rib segmentation defects	Multiple independently reported families with compatible recessive disease [14,34].	*DLL3*/Notch segmentation models support the developmental link.	Established; does not validate every allele or predict uniform severity.
*MESP2*	Biallelic variants; SCDO2 and allelic STD	Distinct human recruitments support the recessive segmentation relationship [11,15,63].	Variant assays and somite boundary biology support loss of function.	Established; founder effects and incomplete genotypes constrain subtype claims.
*LFNG*	Biallelic variants; SCDO3 with vertebral/rib abnormalities	Original and subsequent human families support recessive disease [11,12,41,64].	Localization, enzymatic and expression assays [65] support impaired function.	Established; severity and axial distribution remain variable.
*HES7*	Biallelic variants; SCDO4 with segmentation defects	Original and later families provide compatible human evidence [13,16,66].	Repression/dimerization assays support clock disturbance.	Established; laterality and neural-tube findings are not obligatory.
*DLL1*	Biallelic p.Gly512Arg; recessive vertebral segmentation disorder	Two affected brothers in one family; no independent family replication [39].	Patient-sample Notch readouts support, but do not prove, the mechanism.	Emerging; heterozygous *DLL1* neurodevelopmental disease is a different relationship [38].
*RIPPLY2*	Biallelic variants; cervical/cervicothoracic SCDO6	Separate family reports support compatible recessive disease [35,36,37,67].	Patient-variant repression assays [35] support impaired function; murine boundary-formation studies [68] provide mechanistic context.	Established; regional anatomy varies between reported families.
*PAX1*	Biallelic variants; syndromic otofaciocervical syndrome type 2	Independent affected families show recessive syndrome evidence [55,56].	Transcriptional and sclerotome-development data are supportive.	Established for OFCS2; not transferable to isolated CVM.
*PAX1*	Rare heterozygous coding variants; candidate CVM susceptibility	Two candidate heterozygous variant carriers were identified among 48 patients with CVMs. One carrier also had a ventricular septal defect and polydactyly, and both variants were present in unaffected mothers. The variants were also observed at low frequencies in population data [69].	*Pax1* loss and regulatory-element models support axial relevance [51,52,57].	Hypothesis-generating; replicated human isolated-CVM causality is lacking.
*PAX9*	Proposed human CVM relationship; inheritance undefined	No convincing replicated *PAX9*-CVM human relationship identified.	*Pax1*/*Pax9* animal interaction supports vertebral/disk development [53,54].	Hypothesis-generating; animal synergy cannot establish human CVM diagnosis.
*VANGL1*	Rare hypomorphic/dominant-negative variants; CVM susceptibility	One multicenter study; nine *VANGL1* variants within 19 combined *VANGL1*/2 carriers [23].	Variant assays and Vangl/low-oxygen models support context dependence.	Emerging; shared cohort is not external replication, and variant effects vary.
*VANGL2*	Rare hypomorphic/dominant-negative variants; CVM susceptibility	Same 782-patient study; eight *VANGL2* variants within 19 combined carriers [23].	Cellular and dosage/low-oxygen models support susceptibility.	Emerging; shares the *VANGL1* cohort and lacks full variant-level attribution.
*KAT6B*	Promoter methylation; CS/Cobb-angle association	One 50-case/50-control blood study; no external replication [59].	Facet-joint and chondrocyte experiments support a candidate pathway.	Hypothesis-generating; cross-sectional methylation is not causal or prognostic proof.
*TNS3*	Promoter methylation; congenital scoliosis association	Twin-pair discovery and 50-case/50-control validation within one program [58].	Cartilage staining provides expression support.	Hypothesis-generating; no external replication; cohort overlap with *KAT6B* is possible.
*ITPR2*	Ultra-rare variant burden; CVM risk candidate	One case–control burden analysis; external replication not identified [40].	Developing-spine expression provides biological context.	Emerging; burden does not classify each allele or establish clinical utility.
*TPO*	Ultra-rare variant burden; CVM risk candidate	One case–control burden analysis; external replication not identified [40].	Embryonic-spine expression is contextual, not variant validation.	Emerging; cannot transfer other *TPO* disease relationships to CVM.
*H6PD*	Ultra-rare variant burden; CVM risk candidate	One case–control burden analysis; external replication not identified [40].	Developmental expression/pathway data support plausibility.	Emerging; no established Mendelian CVM mechanism or allele-level diagnosis.
*SEC24B*	Ultra-rare variant burden; CVM risk candidate	One case–control burden analysis; external replication not identified [40].	Trafficking and planar cell polarity biology provide context.	Emerging; functional plausibility does not validate every human variant.
*NADSYN1*	Biallelic variants; congenital NAD deficiency/VCRL spectrum	Unrelated families and later new cases support recessive multisystem disease with vertebral defects [70,71].	Protein/enzyme assays and NAD-pathway models support loss of function.	Established for syndromic biallelic disease; not for isolated CVM or a single VUS.
*NADSYN1*	Heterozygous variants; proposed sporadic CVM susceptibility	Nine patients in one series; parental phase unavailable and variants were VUS [44].	Selected variants impaired protein abundance or enzymatic activity.	Emerging; biallelic syndrome validity cannot upgrade this separate relationship.
*DSTYK*	Proposed human CVM relationship; inheritance undefined	Cited study is developmental zebrafish work, not a human CVM cohort [45].	*Dstyk* disruption caused vertebral defects via altered mTORC1/TFEB.	Hypothesis-generating; model relevance does not establish human disease validity.
*COL11A2*	Rare missense variants; vertebral malformation/CS susceptibility	Three reported human patients across two studies; segregation remains incomplete [46].	Zebrafish loss and failed rescue by variant constructs support plausibility.	Emerging; unaffected carrier and small human series limit penetrance claims.
*FBN1*	Rare variants; CVM/congenital scoliosis-specific relationship	One family plus case–control enrichment in one study [47].	One missense allele increased TGF-beta signaling in vitro.	Emerging; established fibrillinopathy does not validate every CVM allele.
*SOX9*	p.Met469Val; proposed nonsyndromic CVM relationship	Same rare allele in three patients from one study; no external replication [48].	Luciferase assay showed altered transactivation.	Emerging; *SOX9*–campomelic dysplasia validity cannot be generalized to this allele.
(**b**)				
Specified Relationship		Reported Human Data and Source-Defined Pedigree Count	Segregation and Phase	Current Diagnostic and Prognostic Boundary
*TBX6* canonical compound genotype		Wu 2015: 161 discovery cases and 76 additional cases; later independent series [7,8,10].	Rare loss-of-function/deletion and T-C-A haplotype in trans; either component alone is insufficient.	Concordant complete genotype can support etiological diagnosis.
*TBX6* noncanonical biallelic missense		Three non-duplicate missense/missense pedigrees in the reviewed reports: one French-recruited, one Japanese and one Egyptian-ancestry US pedigree [28,61,62]. Risk-haplotype and deletion/missense cases are excluded; exact combinations differ. See Table S7.	Allelic phase and exact combination must be shown; effects differ by allele.	Case-specific research interpretation; not interchangeable with the canonical *TBX6* diagnostic model.
16p11.2 duplication including *TBX6*		Four of seven selected carriers had CVMs involving the cervical spine [29]. Earlier duplication reports described spinal phenotypes, but their comparability with this specific cervical pattern remains uncertain.	Multigene copy-number gain; segregation and population penetrance not estimable.	Interpret the full CNV and phenotype; no *TBX6*-only attribution or prognostic rule.
*DLL3* biallelic SCDO1		Thirteen non-duplicate pedigrees in the two foundational reports: three in Bulman 2000 plus ten explicitly additional families in Turnpenny 2003 [14,34]. This is a source-defined count, not a worldwide census.	Compatible biallelic recessive segregation is reported.	May support SCDO1 diagnosis with concordant alleles and anatomy; no uniform severity prediction.
*MESP2* biallelic SCDO2		Thirteen genotype-complete pedigrees: one in Whittock 2004 plus twelve eligible pedigrees in Cornier 2008 [15,63]. Cornier Table 2 family 10 has only one identified allele and is excluded; related cousins remain one pedigree.	Complete biallelic genotype required; an isolated unresolved allele is insufficient.	May support SCDO2 or allelic STD diagnosis; subtype anatomy is not deterministic.
*LFNG* biallelic SCDO3		Ten non-duplicate reported pedigree/case units in the audited reports [12,41,64,72,73,74,75,76]. One Arab case is re-reported; the two Japanese cases are distinct. Some reported genotypes include VUS and are not all proven molecular diagnoses.	Homozygous or compound-heterozygous variants with compatible phenotype.	May support SCDO3 diagnosis; axial distribution and severity vary.
*HES7* biallelic SCDO4		Five pedigree units in the reviewed reports: one in 2008, one in 2010 and three in 2013 [13,16,66]. The 2013 extended kindred is counted once alongside two unrelated families; seven individuals are not seven pedigrees.	Compatible recessive segregation in reported families.	May support SCDO4 diagnosis; associated laterality or neural-tube findings are not obligatory.
*DLL1* biallelic p.Gly512Arg		One family with two affected brothers; no independent family replication [39].	Both affected homozygous; parents heterozygous in the reported family.	Emerging recessive vertebral relationship; no established standalone diagnostic or prognostic rule.
*RIPPLY2* biallelic SCDO6		Six non-duplicate pedigrees: 1 + 1 + 2 + 2 in McInerney-Leo 2015, Karaca 2015, Serey-Gaut 2020 and Wegler 2021 [35,36,37,67]. Shared alleles alone are not duplicate-patient evidence; splice-allele classification remains separate.	Biallelic recessive genotypes with compatible regional defects; assess each allele separately.	May support a regional segmentation diagnosis; vertebral level and clinical course vary.
*PAX1* biallelic OFCS2		Seven non-duplicate OFCS2 pedigrees across six clinical reports [55,56,77,78,79,80]. Yamazaki 2020 [77] reuses the Paganini 2017 [78] P2/P3 pedigree; this is counted once. Counts concern the syndrome, not seven isolated-CVM pedigrees.	Biallelic recessive variants; seek syndromic craniofacial, shoulder and immune features.	Supports syndrome-specific diagnosis, not a generic isolated-CVM designation.
*PAX1* candidate heterozygous CVM relationship		Two candidate heterozygous variant carriers were identified among 48 patients with CVMs. One carrier also had a ventricular septal defect and polydactyly, and both variants were present in unaffected mothers. The variants were also observed at low frequencies in population data [69].	Same candidate alleles were found in unaffected mothers.	Not a validated isolated-CVM molecular diagnosis.
*PAX9* candidate human CVM		No independent human report meeting the predefined CVM-relationship criteria was identified in the sources audited for this review (count within this audit: 0). This is not a census of all *PAX9* human studies; other *PAX9* disease relationships are separate.	No CVM-specific inheritance or segregation model established.	Developmental candidate only; animal evidence does not establish human CVM diagnosis.
*VANGL1* susceptibility		One multicenter study of 782 cases; nine *VANGL1* variants within 19 combined *VANGL1*/2 carriers [23].	Variant-specific susceptibility; parental data and penetrance are incomplete.	Emerging association, not a validated monogenic or prognostic test.
*VANGL2* susceptibility		Same 782-case study; eight *VANGL2* variants in the shared 19-carrier group [23].	Variant-specific susceptibility; shared cohort is not external replication.	Emerging association, not a validated monogenic or prognostic test.
*KAT6B* methylation association		One 50-case/50-control blood study; no independent replication [59].	Postnatal methylation association, not inherited variant segregation.	Research association only; not a causal diagnostic or prognostic biomarker.
*TNS3* methylation association		Two discordant twin pairs and within-program 50-case/50-control validation [58].	Postnatal methylation, not inherited variant segregation.	Research association only; external replication and causal direction remain open.
*ITPR2* burden candidate		Total cohort: 873 cases/3794 controls; burden analysis: 744 undiagnosed cases/3740 controls passing QC. No external replication identified [40].	Gene-level enrichment; no single validated inheritance model for all alleles.	Candidate susceptibility signal; not an individual-variant diagnosis or prognosis.
*TPO* burden candidate		Same burden study; 12 qualifying carriers among cases and 12 among controls [40].	Gene-level enrichment; thyroid-disease validity cannot transfer to each CVM allele.	Candidate susceptibility signal only.
*H6PD* burden candidate		Same burden study; 11 case carriers and 26 controls [40].	Predominantly heterogeneous heterozygous variants; no general Mendelian CVM model.	Candidate susceptibility signal only.
*SEC24B* burden candidate		Same burden study; nine case carriers and 15 controls [40].	No general Mendelian CVM segregation model established.	Candidate susceptibility signal only.
*NADSYN1* biallelic syndromic disease		Fourteen non-duplicate CNDD pedigrees: four in 2020 plus ten further families in 2024 [70,71]. Vertebral anomalies occur in 3/5 individuals in 2020 and 10/12 with structural anomalies in 2024; this is not a count of fourteen vertebral-positive pedigrees.	Complete biallelic genotype in multisystem congenital NAD-deficiency context.	May support syndrome-specific diagnosis; not an isolated-CVM claim.
*NADSYN1* heterozygous CVM candidate		Nine unrelated patients in one series; no external replication [44].	Parental DNA unavailable; candidate variants were VUS.	No validated heterozygous CVM diagnosis.
*DSTYK* candidate human CVM		Cited study supplied zebrafish developmental evidence, not an independent human CVM cohort [45].	No human CVM-specific segregation model.	Experimental relevance only; no human CVM diagnostic claim.
*COL11A2* missense susceptibility		Three patients across two studies; two shared p.Arg130Trp [46].	One reported unaffected maternal carrier; penetrance and segregation unresolved.	Candidate relationship; no general clinical prediction.
*FBN1* CVM-specific candidate		One segregating family plus case–control enrichment in one study [47].	A truncating allele segregated in one family; effects differ among alleles.	Interpret established fibrillinopathy separately from proposed CVM-specific susceptibility.
*SOX9* p.Met469Val candidate		Three patients carrying the same allele in one study; no independent replication [48].	Family penetrance and segregation not fully resolved.	Candidate allele-specific relationship, not a general *SOX9*-CVM rule.

Tiers apply only to the specified relationship, inheritance and variant mechanism in each row. Established denotes replicated human vertebral evidence with coherent genetic support and no unresolved material contradiction; Emerging denotes direct human germline genetic support with remaining gaps that meets the minimum defined in Section 2 (a significant controlled association, co-segregation in at least one family or functional evidence specific to the patient alteration); Hypothesis-generating denotes evidence below that minimum, including non-genetic methylation associations. These are author-defined review tiers, not ClinGen gene–disease validity or ACMG/AMP variant classifications, and they do not establish prognosis or clinical utility. Table S2 gives study-level detail and three additional broad exploratory categories that are not represented here as single named gene/locus relationships.

Counts in Table 1b are non-duplicate pedigree or case units in the specifically audited primary reports, not an exhaustive worldwide census or counts of independent mutation origins. A sibship is one pedigree; explicitly re-reported cases and functional reuse are not new pedigrees. Complete biallelic findings are distinguished from single unresolved alleles. A counted pedigree does not establish pathogenicity of every variant; *LFNG* reports include VUS, and *PAX1*/*NADSYN1* counts concern syndromic relationships rather than uniformly vertebral-positive families. Table S7 gives all 31 report-level decisions, source locations and arithmetic. Table S2 retains the relationship-specific appraisal. No relationship in Table 1 supports genotype-only prediction of progression, surgical need or functional outcome; the final column therefore records only relationship-specific diagnostic limits.

## 4. Developmental Context for Variant Interpretation

### 4.1. Segmentation-Clock Signaling and Somite Boundaries

Vertebrae arise from paraxial mesoderm through periodic somite formation, followed by regionalization, sclerotome differentiation, resegmentation, chondrogenesis and ossification [17,18]. Somitogenesis establishes the repeated units of the axial skeleton and depends on an oscillatory segmentation clock coupled to a moving determination front.

Notch, Wnt and FGF signaling coordinate oscillation and positional information in presomitic mesoderm [18,81,82,83]. Human pluripotent-stem-cell systems reproduce features of paraxial mesoderm differentiation, clock oscillation and epithelial somite formation [84,85,86,87,88]. Qin and colleagues instead directly reprogrammed urine-derived cells into induced presomitic mesoderm; this is a distinct human cell-based model, not an hPSC differentiation experiment [89]. Clock periodicity, synchronization and boundary readouts offer complementary assays of candidate perturbations. Mechanical regulation in experimental somitogenesis adds a tissue-level context without establishing a human gene–disease diagnosis [90,91,92].

The human disorders caused by *DLL3*, *MESP2*, *LFNG* and *HES7* demonstrate that perturbing this system produces vertebral and rib segmentation abnormalities. FGF-dependent regulation of *HES7*, *LFNG* dosage and direct measurements of clock synchronization further indicate that pathway effects are networked rather than linear [93,94,95,96]. Clinically observed block vertebrae or multilevel segmentation defects may reflect failed boundary formation, but similar anatomy can also arise through later morphogenetic disturbances.

Clock and wavefront models also make timing mechanistically relevant. A transient perturbation may affect only a restricted axial level, whereas persistent disruption can produce extensive vertebral and rib involvement. Variation in oscillation period, tissue synchronization, stochastic gene expression, environmental stress or the position of the determination front may therefore contribute to differences in defect number and distribution [97]. Human imaging cannot directly reconstruct these embryonic dynamics, but detailed mapping of axial patterns can nevertheless generate hypotheses for experimental testing [18,98].

A translational limitation is that postnatal imaging captures the final structure rather than the embryonic event. A formation defect, segmentation defect or mixed pattern should be treated as a developmental hypothesis, not a one-to-one molecular diagnosis. Genotype–phenotype studies are more informative when imaging labels are combined with vertebral level, rib pattern, symmetry, associated anomalies and three-dimensional morphology.

### 4.2. Sclerotome Specification, Resegmentation and Disk Development

After somite formation, ventromedial cells acquire sclerotome identity under notochord and floor-plate signaling. *Pax1*/*Pax9* experimental studies support roles in vertebral body and intervertebral disk development; a blanket attribution of neural-arch defects to these factors is not justified [17,53,54,99]. SHH signaling, *SOX9*-dependent chondrogenesis and extracellular matrix programs are relevant to differentiation, but their experimental roles and human disease relationships require separate appraisal.

Resegmentation requires coordinated separation and recombination of adjacent sclerotome domains. Disturbance at this stage may affect vertebral body shape, disk boundaries, spinal-nerve patterning and the relationship between vertebrae and ribs even when initial somite periodicity is preserved [100,101]. Experimental loss of *Pax1*, particularly together with *Pax9* deficiency, produces severe axial skeletal and disk abnormalities [52,53].

Notochord–sclerotome interactions also shape the vertebral-disk unit. Sclerotome cells form the annulus fibrosus and vertebral structures around notochord-derived nuclei pulposi, so defects in cell identity, condensation or extracellular matrix organization can alter both vertebral morphology and disk boundaries. A recent synthesis of amniote sclerotome development provides a detailed framework for these transitions [102]. These later processes may account for phenotypes that do not fit a pure somite boundary defect, although direct human mechanistic evidence remains sparse [51,54,103].

This distinction refines interpretation: clock–boundary programs establish repeated axial units, whereas sclerotome programs contribute to vertebral and disk identity. The strong experimental evidence for both *Pax1* and *Pax9* does not justify a single human CVM grade for *PAX1*/*PAX9*. *PAX1*-related otofaciocervical syndrome and candidate heterozygous PAX1–CVM associations must remain separate [55,56].

### 4.3. Tissue Polarity, Morphogenesis and Regulatory Modulation

The vertebral column is a three-dimensional structure that requires coordinated cell polarity, migration, condensation and growth. Planar cell polarity signaling contributes to oriented cell behavior and tissue architecture, and human genetic evidence implicating *VANGL1*/*VANGL2* in CVMs suggests that morphogenetic coordination represents a distinct mechanistic layer [23]. Zebrafish planar cell polarity models also connect congenital scoliosis-like phenotypes to altered notochord morphogenesis [104]. Interactions among Wnt, FGF, Notch, SHH, extracellular matrix and mechanical signals may account for why different molecular disturbances converge on overlapping anatomy.

Histone methylation regulates transcription and differentiation in skeletal and neural developmental contexts [105,106]. These findings provide general biological context rather than direct evidence for a causal chromatin mechanism in human CVMs. Accessible-tissue DNA-methylation studies in congenital scoliosis cannot reliably distinguish primary developmental causes from secondary or compensatory signatures [58,60].

Developmental pathways are better interpreted as interacting modules rather than isolated linear cascades. Planar cell polarity can influence tissue movement while canonical Wnt, FGF and Notch signals regulate patterning and timing; SHH and PAX programs then shape sclerotome fate. Evidence from related neural-tube and axial-patterning disorders indicates pathway overlap, but phenotype transfer across disorders must remain cautious [23,107]. A systems view remains useful only if it preserves gene-specific evidence strength.

Maternal and environmental factors belong in the proposed context but require their own evidentiary bounds. Gestational hypoxia interacts with genetic susceptibility in experimental somitogenesis [24]; the Vangl model similarly demonstrates context-dependent vertebral defects in mice [23]. Maternal diabetes and teratogenic exposures have been discussed among human CVM risk factors but do not establish one measured threshold for an individual pregnancy [2]. Exposure history should be considered without assigning causation from retrospective association. Somatic mosaicism could create spatially restricted perturbation, yet routine blood sequencing cannot test embryonic axial mosaicism directly; this remains a hypothesis rather than an established explanation for unresolved CVM. Epigenetic cause, modifier and consequence are likewise separate alternatives requiring temporal and tissue-specific evidence.

CVM mechanisms can thus be organized across four interacting levels: paraxial mesoderm specification, somite boundary formation, sclerotome and vertebral-disk differentiation, and three-dimensional tissue morphogenesis. This modular organization is more informative than a flat gene list: it links molecular findings to testable developmental processes while preserving uncertainty about emerging candidates.

### 4.4. A Spatiotemporal Developmental-Threshold Model of CVM

Here, a developmental threshold is defined operationally as an assay-specific perturbation level at which the probability of a prespecified abnormal axial-development readout exceeds a prespecified biologically meaningful level. The input might be gene dosage, perturbation duration or exposure intensity; the output might be the proportion of intact somite boundaries. Lineage, developmental interval and fraction of tissue exposed condition that input–response relationship rather than constituting one universal cutoff. This statistical boundary need not imply an abrupt molecular switch. No numerical value has been estimated for human embryos, and postnatal imaging cannot recover one. The framework is a proposed conceptual synthesis, not a validated diagnostic or prognostic tool.

Human observations directly support specified disease relationships, including *TBX6* compound inheritance and vertebral phenotypes [7,8,10]; they do not measure a developmental threshold. Experimental antecedents include dose- and environment-dependent vertebral effects in Vangl models and a hypoxia–Notch interaction affecting somitogenesis [23,24]. Human stem-cell systems permit measurement of clock dynamics and boundary formation [84,85,88], but their assay responses have not established a numerical threshold in affected patients. The claim that the same threshold logic explains different anatomical distributions of CVM is therefore an extrapolation to be tested, not a demonstrated human result.

The specific hypothesis is that, within a defined developmental module, perturbation timing, duration and spatial extent alter the frequency and axial distribution of malformed units. Neither a formation/segmentation label nor a later curve angle identifies the embryonic window. Table S6 pairs each relative developmental interval with its tissue, molecular process, expected pattern, measurable readout and relationship-specific human-evidence boundary; it does not assign unverified human gestational dates or claim that one imaging pattern uniquely diagnoses one mechanism.

Developmental transcriptomic mapping of genetic burden signals can prioritize candidate cell states but does not prove their causal order in patients [40]. Section 6.2 and Section 7 propose tests of particular stage-specific relationships and identify observations that could support, weaken or leave those relationships unresolved.

## 5. Phenotype-Led Genomic Diagnosis

### 5.1. Standardized Phenotyping for Genomic Interpretation

Imaging defines the structural phenotype and its clinical course. Radiographs document balance and progression; CT or three-dimensional reconstruction can clarify complex osseous anatomy when the information changes management, and MRI evaluates neural-axis abnormalities. Congenital scoliosis series and meta-analysis describe associated vertebral, rib and neural findings [108,109,110,111]. The two Lin reports analyze the same surgical cohort rather than independent replication [112,113]. Referral and operative selection can enrich severe or associated phenotypes, so these studies do not estimate anomaly prevalence in all CVMs.

Mechanism-informed phenotyping records morphology without treating it as a one-to-one molecular readout [3,6]. Cardiac–vertebral co-occurrence is supported by congenital-deformity cohorts and meta-analysis [114,115]. Shared developmental signaling provides a possible biological context, but co-occurrence does not establish a shared causal gene in an individual. Alagille syndrome illustrates a supported syndromic relationship: *JAG1*/*NOTCH2*-related disease can combine butterfly vertebrae and cardiac abnormalities [116]. The 22q11.2 deletion syndrome offers a chromosomal example of multisystem assessment rather than proof that one locus explains all cardiac–vertebral combinations [43]. *HES7*-related SCDO with dextrocardia and situs inversus has also been reported; laterality disturbance should not be conflated with all structural congenital heart disease [66].

Quantitative three-dimensional phenotyping can add vertebral shape and orientation to curve-angle labels. In a hemivertebra-specific biplanar-radiography study, reconstructed vertebral shape and pose, global posture and imbalance provided several measurable descriptors, with CT-based reconstruction used for comparison [117]. These measurements illustrate how the location and geometry of an individual malformed vertebra can be recorded alongside whole-spine alignment. They may improve anatomical characterization and help define the question for clinical imaging, but this small selected series does not validate every mixed defect or show that imaging measurements alone determine management. Protocols should prespecify vertebral level, wedge geometry, rotation, posterior elements, rib pattern, measurement reliability and missingness. Clinically indicated images should be reused where possible rather than adding radiation solely for research phenotyping.

Phenotype quality determines whether genetic associations are interpretable. Broad congenital scoliosis labels can mix isolated formation defects, multilevel segmentation disorders and syndromic cases, obscuring relationship-specific signals. Prespecified quantitative descriptors may improve stratification and genotype–phenotype comparison by reducing misclassification and permitting shared variables across centers. This is a methodological expectation, not demonstrated improvement in gene-discovery yield. Discovery studies should assess missingness and interobserver agreement, distinguish severity from mechanism, and test replication within the same phenotype definition before proposing new CVM genes.

Computational image analysis may assist structured phenotype extraction. Available scoliosis work includes idiopathic, early-onset and mixed spine populations [118,119,120,121]. CVM-specific labels, congenital subgroup performance and external validation remain necessary before these tools support independent clinical decisions.

### 5.2. Prenatal and Postnatal Genomic Evaluation

Prenatal ultrasound is the first-line modality for detecting fetal vertebral and rib abnormalities and associated malformations [122,123]. Fetal MRI can provide additional anatomical information in selected complex cases [124]. Historical and contemporary fetal cohorts indicate that outcome depends largely on whether the vertebral defect is isolated or accompanied by other abnormalities [125,126,127]. However, imaging alone rarely establishes molecular etiology or reliably predicts postnatal progression, particularly for an apparently isolated hemivertebra [128,129]; a systematic review has specifically addressed genetic etiologies and outcome in this isolated presentation [130].

Prenatal testing is selected according to the complete fetal phenotype and counseling, not a universal sequence of assays. Microarray addresses chromosomal imbalance; phenotype-informed sequencing may be considered for a suspected monogenic condition, particularly when conventional testing is unrevealing [131]. Broader fetal structural-anomaly studies support context-dependent use of trio sequencing but do not establish CVM-specific yields [132,133,134]. Table 2 presents options and evidentiary limits, not a recommendation to perform microarray followed by exome in every case.

**Table 2:** Phenotype-guided molecular evaluation of congenital vertebral malformations.

Phenotypic Context	Possible Testing Options	Interpretation and Family Work	Evidence Boundary
Isolated formation defect	Genetics evaluation when context supports it; microarray, directed panel or ES/GS selected case by case	Survey associated anomalies; parental/phase testing if a candidate arises	No fixed sequence or transferable pooled yield for all isolated CVMs [128]
Multilevel vertebral–rib segmentation	SCDO-focused panel or broader sequencing; CNV assessment as appropriate	Evaluate recessive alleles, segregation and disease-specific anatomy	Relationship validity differs from individual variant classification [11]
Multisystem anomalies/developmental disability	Microarray and/or ES/GS based on phenotype and local pathway	Whole-phenotype assessment; trio analysis where useful	Broad pediatric guideline supports ES/GS; not an isolated-CVM yield estimate [135]
Familial or consanguineous presentation	Phenotype-informed sequence/CNV testing with relatives	Phase, segregation, penetrance and inheritance evaluation	Unaffected carriers may be compatible with susceptibility or reduced penetrance
Prenatal vertebral abnormality	Expert fetal imaging; chromosomal assessment; sequencing for selected suspected monogenic cases	Prenatal counseling, parental samples and complete fetal phenotype	ISPD guidance is broader than CVM; selected fetal cohort denominators matter [131]
Unresolved or VUS result	Phenotype update and periodic reanalysis; additional testing based on unresolved mechanism	Reclassification through clinical laboratory/genetics team	No surgery or altered surveillance based on VUS alone [26]
Regulatory or mosaic hypothesis	Specialist/research lineage, tissue or functional studies when appropriate	Blood-negative finding does not test every embryonic tissue	Generally not routine CVM testing; do not substitute research plausibility for diagnosis

These are phenotype-dependent options, not a fixed testing sequence. Clinical genetics input, counseling, test performance and availability determine their use. Guidelines for broad pediatric anomalies or fetal sequencing should not be interpreted as CVM-specific outcome evidence [131,135].

Postnatal multisystem anomalies, developmental concerns, family history and characteristic vertebral–rib patterns support clinical genetics evaluation. The ACMG pediatric ES/GS guideline applies to congenital anomalies and developmental or intellectual disability broadly, not exclusively to isolated CVMs or prenatal care [135]. Depending on phenotype and local practice, testing can include microarray, a directed panel, exome or genome sequencing. Parental testing can establish phase, de novo occurrence or segregation; periodic reanalysis may revisit unresolved findings as phenotype and knowledge evolve. Tissue-specific mosaic or regulatory assays and experimental rescue generally require specialist or research pathways, rather than being routine CVM tests.

Observed diagnostic yields depend on the tested population and assay. In selected fetal hemivertebra subsets reported by Zhou and colleagues, CMA identified diagnoses in 4/35 isolated cases (11.4%) and 2/23 non-isolated cases (8.7%); after normal CMA results, selected WES subsets yielded diagnoses in 1/9 isolated cases (11.1%) and 2/7 non-isolated cases (28.6%) [128]. These percentages describe tested subsets, not all fetuses in the original cohort.

Other fetal vertebral-anomaly series report results for their overall prenatal testing pathways. Hu and colleagues reported diagnoses in 7/33 isolated cases (21.2%) and 3/19 non-isolated cases (15.8%) using chromosomal analysis and selective WES [123]. Xie and colleagues reported 2/8 (25.0%) and 3/4 (75.0%), respectively, using CMA and additional WES after negative CMA findings [122]. These overall-pathway results are not WES-only yields. None of the three studies reported a statistically significant isolated-versus-non-isolated difference; the non-isolated group in the Xie series contained only four fetuses.

Table S3 preserves the source definitions of isolated and non-isolated phenotypes, detection methods and denominators. Non-isolated fetal vertebral findings are not automatically equivalent to all multisystem CVMs. The studies are not pooled: small samples, referral selection and differences in testing eligibility limit extrapolation to the full CVM spectrum, and non-significant comparisons do not establish equivalent yields. Broader fetal skeletal-anomaly estimates are not isolated-CVM estimates [133,136]. Syndromic diagnoses, including Robinow syndrome with vertebral findings, emphasize whole-phenotype interpretation [137].

A molecular diagnosis can support recurrence-risk counseling and syndrome-specific evaluation but requires concordance across relationship validity, allele classification, inheritance and phenotype. Sequence variants use ACMG/AMP principles; constitutional CNVs use their dedicated standards [26,27]. A VUS is not an independent indication for surgery, altered surveillance or predictive family testing. Additional phenotypic, segregation or functional information may prompt reclassification; anatomy and established clinical indications remain the basis of care.

## 6. Evidence-Bounded Genotype–Phenotype Interpretation

### 6.1. Clinical Interpretation Within Evidence Boundaries

Stratification should integrate structural phenotype, molecular etiology, developmental mechanism, associated anomalies, family history and longitudinal outcome. The aim is to refine etiological interpretation and research grouping, not to replace morphology-based care. Patients with an isolated hemivertebra and those with extensive rib–vertebral segmentation defects may share a clinical label while differing substantially in mechanism and recurrence risk.

Current clinical evidence supports etiological clarification and recurrence-risk counseling after an adequately supported molecular diagnosis. Mechanism-aware phenotyping and harmonized categories may also improve research stratification and multicenter cohort design, but these are proposed methodological benefits rather than demonstrated gains in gene-discovery yield or clinical outcomes. Evidence for using genotype alone to forecast progression, operative timing or long-term function remains limited. Preliminary TACS outcome studies also require external replication before changing management [33].

Figure 4 separates the type of alteration from its clinical classification: a CNV can be pathogenic, likely pathogenic or uncertain, just as a sequence variant can. A negative result is a testing outcome, not a variant class. Relationship validity, inheritance, molecular mechanism and phenotype concordance are then considered together. P/LP status alone does not establish that a finding explains the vertebral phenotype, and the review’s relationship tiers do not replace laboratory classification.

### 6.2. Testable Predictions and Falsification Criteria

For each proposed test, preregister the variant or exposure, lineage and stage definition, independent biological replicate unit, primary readout, assay-quality criteria, sample-size rationale, and a numerical negligible-effect margin (Δ) appropriate to that assay. Report effect sizes and confidence intervals, including failures of model qualification. These experiment-specific decision rules make a particular claim testable; no universal Δ or embryonic threshold is inferred from the review.

Prediction 1 tests timing and spatial extent within one defined module. In an isogenic axial model, apply matched effective perturbations during prespecified stage windows and, where feasible, to restricted versus widespread cell populations. The primary outcome is the proportion and axial position of boundaries failing a predefined morphology criterion per independent differentiation, not the later curve angle. Support requires a reproducible stage-by-perturbation or area-by-perturbation effect whose confidence interval excludes the prespecified negligible-effect range (±Δ). An adequately precise effect confined within ±Δ after assay-quality checks weakens this specific prediction; insufficient perturbation, poor lineage identity or imprecise estimates leave it unresolved.

Prediction 2 tests whether an assay-specific response boundary or modifier interaction exists, not whether every CVM mechanism is nonlinear. Quantify perturbation dose and the fraction of intact boundaries across a prespecified dose series and genetic or environmental backgrounds [7,8,23,24]. Estimate the dose at which an abnormal-readout probability crosses a prespecified biological criterion, with a confidence interval; compare a change-point model with a smooth continuous model on held-out differentiations. Support for a sharp response requires reproducible out-of-sample improvement over the smooth model; a modifier claim additionally requires a replicated interaction exceeding its prespecified negligible-effect range. If a qualified, sufficiently precise smooth model fits equally well and no material interaction is observed, those specific claims are weakened. A smooth response may still cross a decision boundary and is not evidence for a universal human cutoff.

Prediction 3 tests a specified candidate allele in a qualified, stage-matched human axial lineage. Before analysis, choose the relevant primary endpoint—clock period/coherence, boundary integrity, sclerotome identity or matrix organization—and define assay-quality and negligible-effect margins [84,85,88]. An allele effect replicated across independent differentiations, with correction or mechanism-specific rescue exceeding the prespecified margin while viability and cell-state composition remain comparable, supports that allele–process link. An adequately precise null effect within the margin, or failure of a verified rescue, weakens that link; an underpowered or failed model is inconclusive. Functional results do not by themselves establish a human gene–disease relationship or genotype-only prognosis.

## 7. Limitations of This Review and Research Priorities

### 7.1. Limitations of the Review

The review is a selective narrative synthesis. Although the final original search dates, absence of language restrictions and eight PubMed/MEDLINE strategies have been recovered, the full search history and original identification, deduplication, screening, exclusion and inclusion counts remain unavailable. Searches of databases other than PubMed/MEDLINE were supplementary, and their search terms were not retained. Literature screening was not independently duplicated. Revision-stage tier assignments were independently reassessed by Qi Zhang, with agreement on all 29 records; this does not remedy single-author source selection or constitute a formal reliability study. Neither formal risk-of-bias assessment nor a quantitative evidence score was used. Revision-stage retrieval does not eliminate these limitations; it was confined to PubMed-indexed material plus targeted source checking, and one broad query was capped. Phenotype definitions, assay eligibility and referral settings vary, some publications reuse cohorts, and small families or candidate reports can dominate particular relationships. Publication and language/accessibility bias are possible but unquantified. Evidence tiers remain judgments with explicit criteria rather than validated scores, and recent publications may change the appraisal. The conceptual model has not been calibrated against human embryonic measurements or prospectively validated clinical outcomes.

### 7.2. Priorities for Validation

Inconsistent terminology continues to hinder interpretation. Congenital scoliosis, CVMs and syndromic segmentation disorders are often grouped despite different inclusion criteria. Studies should define the structural phenotype first and then report the clinical deformity, associated anomalies and molecular context. This distinction should improve reproducibility and reduce inappropriate transfer of evidence between disease groups.

A second priority is functional validation. Sequencing can identify variants and burden signals, but causal interpretation requires developmentally relevant models. Patient-derived induced pluripotent stem cells, sclerotome-like differentiation, organoids, zebrafish and mouse models, and targeted perturbation can be used to test whether candidate genes alter somite periodicity, boundary formation, sclerotome identity or vertebral morphogenesis [17,18].

Multi-omic studies also require stronger developmental anchoring. Epigenomic or transcriptomic signatures are informative only when linked to a relevant cell population, developmental window and experimentally testable pathway. Small studies of blood or surgical tissue should not be interpreted as direct readouts of early embryonic causation.

Selective publication and narrative inclusion may favor coherent positive findings, but the magnitude of publication bias in this review has not been measured. Reporting negative results, prespecifying phenotype definitions, separating discovery from validation and disclosing cohort overlap would improve appraisal [19,20].

Longitudinal genotype–phenotype cohorts are also needed. Cross-sectional genetic discovery cannot establish whether a molecular subtype adds predictive value beyond anatomy, age and growth. Prospective multicenter studies should combine standardized three-dimensional imaging, neural-axis and systemic phenotypes, molecular data, treatment, functional outcomes and patient-reported measures. A long-term follow-up study of patients with congenital scoliosis reported functional outcomes in adulthood after spinal fusion [138].

Multimodal computational tools should be developed only after labels and outcomes are harmonized. Rare, heterogeneous disorders are particularly prone to overfitting. External validation, transparent feature definitions and comparison against conventional clinical variables are required before such models can inform individual decisions.

## 8. Conclusions

CVMs comprise heterogeneous structural outcomes of axial development. Disease-specific genetic evidence is strongest for defined Mendelian relationships and recurrent dosage mechanisms; pathway experiments and candidate associations answer different questions. Spatiotemporal threshold reasoning provides a proposed context for those observations, building on prior developmental and gene–environment models rather than establishing a universal human threshold.

The review’s author-defined hierarchy makes the evidence boundary explicit: molecular diagnosis depends on a supported relationship, a compatible allele and inheritance pattern, and the patient’s phenotype. Quantitative imaging and stage-matched functional studies may improve future stratification, but prospective and diverse-ancestry validation remains necessary before molecular categories add reliable clinical prediction.

## Data Availability

No new patient or experimental data were generated. Recovered original search strategies, documented revision-stage retrieval counts and evidence-appraisal summaries are provided in the Supplementary Materials. Original retrieval and study-selection counts were not retained.

## Supplementary Materials

The following supporting information can be downloaded at: https://www.mdpi.com/article/10.3390/genes17101309/s1. Tables S1–S7 provide recovered original and documented revision-stage retrieval strategies, detailed relationship appraisal, assay-specific prenatal yields, comparison with prior work, tier criteria and developmental-stage mapping. Table S1a. Search provenance, eligibility and study selection. Table S1b. Exact revision-stage PubMed-indexed retrieval strategies. Table S1c. Recovered original PubMed MEDLINE search strategies. Table S2a. Relationship-specific evidence assessment for genes and mechanisms discussed in the review. Table S2b. Condition-by-condition record for the 29 Table S2a entries. Table S3. Context specific prenatal diagnostic yields. Table S4. Comparison with prior reviews and models. Table S5. Operational evidence tier criteria. Table S6. Developmental windows, testable mechanisms and human-evidence boundaries. Table S7a. Source-defined pedigree counts for Table 1b. Table S7b. Primary report audit and duplicate decisions.

## Abbreviations

AR, autosomal recessive; AD, autosomal dominant; ACMG, American College of Medical Genetics and Genomics; AMP, Association for Molecular Pathology; ClinGen, Clinical Genome Resource; CMA, chromosomal microarray analysis; CNV, copy-number variant; CVM, congenital vertebral malformation; ES/GS, exome/genome sequencing; FGF, fibroblast growth factor; hPSC, human pluripotent stem cell; ISPD, International Society for Prenatal Diagnosis; PCP, planar cell polarity; P/LP, pathogenic/likely pathogenic; PSM, presomitic mesoderm; SCDO, spondylocostal dysostosis; SV, structural variant; TACS, *TBX6*-associated congenital scoliosis; VUS, variant of uncertain significance.
