# Supplementary material for: An Evidence-Tiered Developmental Framework for Congenital Vertebral Malformations: Spatiotemporal Thresholds in Genotype–Phenotype Interpretation
Source: Genes (Basel). 2026 Oct 21;17(10):1309. doi: 10.3390/genes17101309 (PMC13704003; doi:10.3390/genes17101309)
Supplement: Supplementary file 1 [file genes-17-01309-s001.zip › Supplementary Table S2.pdf]

Supplementary Table S2 Evidence Assessment

Relationship appraisal and condition records for 29 entries

Scope. Rows 1–14, 17–18 and 20–29 evaluate named relationships; rows 15, 16 and 19 are broad exploratory observation categories, not gene–disease validity grades for any individual locus or allele. Established requires direct human vertebral evidence, qualifying independent non-overlapping human data beyond discovery, coherent genetic support and no unresolved material contradiction. Emerging records direct human genetic support with at least one established condition unmet or incompletely resolved; its minimum is a significant controlled association, co-segregation in at least one family or functional evidence specific to the patient alteration. Table S2b maps the four conditions to the same 29 entries. Hypothesis-generating relies mainly on exploratory, indirect or model-based evidence; methylation associations cannot exceed this tier. No universal affected-family count is imposed. Unreported segregation, penetrance or expressivity is not treated as negative evidence.

Assessment and interpretation. Xuhao Wang made the initial tier assignments. Qi Zhang independently reassessed all 29 appraisal entries (26 named relationships and three exploratory categories) against the criteria in use at that stage; all 29 assignments agreed, so no disagreement required adjudication. The original literature search and screening were performed by Xuhao Wang alone. These author-defined tiers are not formal ClinGen categories, ACMG/AMP sequence-variant or ACMG/ClinGen CNV classifications, or measures of prognosis or clinical utility.

Table S2a. Relationship-specific evidence assessment for genes and mechanisms discussed in the review

Specified relationship and phenotype	Human evidence and replication boundary	Inheritance and segregation	Functional or developmental evidence	Key limitations and clinical boundary	Tier
***TBX6* canonical compound inheritance CVM / congenital scoliosis**	Wu 2015 reported a discovery series (161 cases; 17 qualifying combinations) and an additional series (76 cases; 6 combinations). Takeda 2017 and later international cohorts supported the specified rare loss-of-function plus T-C-A relationship. Reused or phase-uncertain records were not added as independent cases. [8, 10, 31]	Rare *TBX6* loss-of-function allele, including 16p11.2 deletion, plus the T-C-A hypomorphic haplotype in trans. Neither component alone is treated as sufficient.	Reporter and dosage studies support reduced activity of the hypomorphic background; human and model data support dosage sensitivity.	Variable expressivity and incomplete penetrance preclude deterministic diagnosis or genotype-only prognosis. This row already includes the two 5′ non-coding SNPs in T-C-A and is not duplicated under non-coding variation.	**Established**
***TBX6* noncanonical biallelic missense combinations vertebral and rib segmentation abnormalities**	Three non-duplicate missense/missense pedigrees in the reviewed reports: one French-recruited, one Japanese and one Egyptian-ancestry US pedigree [28, 61, 62]. Risk-haplotype and deletion/missense cases are excluded; exact combinations differ. See Table S7.	Biallelic combinations outside the canonical loss-of-function plus T-C-A model; phase and exact allele pairing are required.	Variant-specific transcription, localization and patient-derived cellular assays provide heterogeneous, assay-dependent support.	Different combinations are not assumed to share one validated mechanism; their biological comparability must be demonstrated before combining their evidence. Normal transcription in one assay and abnormal localization in another are not necessarily contradictory. Complex cases may have additional variants.	**Emerging**
**16p11.2 duplication including *TBX6* cervical CVM susceptibility**	Four of seven selected carriers had CVMs involving the cervical spine [29]. Earlier duplication reports described spinal phenotypes, but their comparability with this specific cervical pattern remains uncertain.	Multigene copy-number gain; proposed *TBX6* dosage increase. Segregation and penetrance cannot be estimated from the selected series.	*Tbx6* overexpression models support dosage plausibility.	The CNV contains multiple genes, so the human phenotype cannot be assigned to *TBX6* alone. Four of seven is not a population penetrance estimate.	**Emerging**
***DLL3* biallelic variants SCDO1**	Thirteen non-duplicate pedigrees in the two foundational reports: three in Bulman 2000 plus ten explicitly additional families in Turnpenny 2003 [14, 34]. This is a source-defined count, not a worldwide census.	Autosomal recessive; biallelic function-disrupting variants with compatible segregation in the disease context.	Human genetic evidence is supported by *DLL3*/Notch segmentation biology and developmental models; not every allele has equivalent direct functional testing.	The established etiological relationship does not imply complete penetrance, uniform severity or allele-specific prognosis.	**Established**

Specified relationship and phenotype	Human evidence and replication boundary	Inheritance and segregation	Functional or developmental evidence	Key limitations and clinical boundary	Tier
***MESP2* biallelic variants**<br>**SCDO2 and allelic spondylothoracic dysostosis (STD)**	Thirteen genotype-complete pedigrees: one in Whittock 2004 plus twelve eligible pedigrees in Cornier 2008 [15, 63]. Cornier Table 2 family 10 has only one identified allele and is excluded; related cousins remain one pedigree.	Autosomal recessive. Single unresolved alleles are excluded from complete relationship evidence.	Variant-level reporter evidence supports loss of *MESP2* function and disturbed somite-boundary specification.	Founder effects and related families limit counts of independent mutation origins. The etiological spectrum is established, but deterministic allele-to-radiographic-subtype prediction is not.	**Established**
***LFNG* biallelic variants**<br>**SCDO3**	Ten non-duplicate reported pedigree/case units in the audited reports [12, 41, 64, 72, 73, 74, 75, 76]. One Arab case is re-reported; the two Japanese cases are distinct. Some reported genotypes include VUS and are not all proven molecular diagnoses.	Autosomal recessive; homozygous or compound-heterozygous variants with compatible vertebral/rib phenotypes.	Variant-specific localization, enzymatic and expression assays support loss of function; Wengryn 2024 is a functional reappraisal, not a new family. [65]	Some published genotypes include VUS; reported-pedigree counts do not confer P/LP status. The two Japanese *LFNG* cases have different ages and alleles. Functional reappraisal adds no human pedigrees. Evidence grade refers to the disease relationship, not every variant.	**Established**
***HES7* biallelic variants**<br>**SCDO4**	Five pedigree units in the reviewed reports: one in 2008, one in 2010 and three in 2013 [13, 16, 66]. The 2013 extended kindred is counted once alongside two unrelated families; seven individuals are not seven pedigrees.	Autosomal recessive; compatible homozygous/biallelic variants and segregation.	Mutant repression and dimerization assays support impaired *HES7* function and segmentation-clock disturbance.	Laterality and neural-tube findings are variably expressed; dextrocardia with situs inversus occurred in only three of seven affected individuals in the 2013 report.	**Established**
***DLL1* p.Gly512Arg biallelic relationship vertebral segmentation disorder / SCDO7**	Barhoumi 2019 reported one consanguineous family with two affected homozygous brothers and heterozygous parents. No independent family repeating the same recessive vertebral relationship was identified. [39]	Autosomal recessive in the reported family; compatible intrafamilial segregation.	Patient-sample Notch-related transcriptional and phosphorylation changes are supportive but not complete variant-mechanism validation.	Replication is insufficient. Heterozygous *DLL1* neurodevelopmental disease is a separate relationship and cannot upgrade the recessive vertebral association.	**Emerging**
***RIPPLY2* biallelic variants**<br>**cervical / cervicothoracic segmentation disorder / SCDO6**	Six non-duplicate pedigrees: 1 + 1 + 2 + 2 in McInerney-Leo 2015, Karaca 2015, Serey-Gaut 2020 and Wegler 2021 [35, 36, 37, 67]. Shared alleles alone are not duplicate-patient evidence; splice-allele classification remains separate.	Autosomal recessive; homozygous or compound-heterozygous variants with compatible family segregation.	A patient truncating variant showed impaired transcriptional repression in a cell assay [35].	Phenotype terminology and axial distribution vary. Relationship validity does not establish genotype-only severity prediction or settle every splice-variant classification.	**Established**
***PAX1* biallelic variants**<br>**otofaciocervical syndrome type 2**	Seven non-duplicate OFCS2 pedigrees across six clinical reports [55, 56, 77, 78, 79, 80]. Yamazaki 2020 [77] reuses the Paganini 2017 [78] P2/P3 pedigree; this is counted once. Counts concern the syndrome, not seven isolated-CVM pedigrees.	Autosomal recessive syndromic relationship; phenotype concordance includes craniofacial, branchial, shoulder-girdle and immune features.	Transcriptional assays and *Pax1* developmental models support impaired sclerotome/axial development.	Established for OFCS2, not for isolated or nonsyndromic CVM. The syndrome evidence must not be transferred to a patient with only a hemivertebra.	**Established**
***PAX1* candidate heterozygous**<br>**CVM relationship**	Two candidate heterozygous variant carriers were identified among 48 patients with CVMs. One carrier also had a ventricular septal defect and polydactyly, and both variants were present in unaffected mothers. The variants were also observed at low frequencies in population data [69].	No coherent replicated human inheritance model for isolated CVM.	*Pax1* loss and regulatory-element models support axial developmental relevance. [51, 52, 57]	Animal and adolescent-idiopathic-scoliosis regulatory data do not establish human congenital-CVM causality. Direct replicated human support is lacking.	**Hypothesis-generating**

Specified relationship and phenotype	Human evidence and replication boundary	Inheritance and segregation	Functional or developmental evidence	Key limitations and clinical boundary	Tier
***PAX9* variation human CVM**	No convincing replicated human *PAX9*-CVM relationship was identified in the current source set. The established human *PAX9* relationship is predominantly tooth agenesis.	No CVM-specific human inheritance or segregation model established.	*Pax1*/*Pax9* animal interaction supports vertebral-body and intervertebral-disc development. [53, 54]	Animal synergy and tooth-agenesis families cannot be transferred to human CVM diagnosis.	**Hypothesis-generating**
***VANGL1* rare hypomorphic or dominant-negative variants CVM susceptibility**	Feng 2024 analyzed 782 patients and reported nine *VANGL1* and eight *VANGL2* rare missense variants in 19 total carriers. Recurrent *VANGL1* alleles were observed, but participating centers in one study are not external replication. [23]	Variant-specific susceptibility with incomplete penetrance and potential background/environment dependence; family information is incomplete for some cases.	Cellular assays and Vangl dosage/low-oxygen mouse models support susceptibility; not every variant was abnormal in every assay.	This row shares the same human study and total carriers with *VANGL2*. Complex phenotypes may have alternative explanations; no general monogenic diagnosis or quantitative human threshold is established.	**Emerging**
***VANGL2* rare hypomorphic or dominant-negative variants CVM susceptibility**	Same Feng 2024 multicenter study and shared set of 19 total *VANGL1*/*VANGL2* carriers; eight *VANGL2* rare missense variants were reported. [23]	Variant-specific susceptibility with incomplete penetrance and potential background/environment dependence.	Cellular assays and Vangl dosage/low-oxygen mouse models support susceptibility.	The *VANGL1* and *VANGL2* rows are not two independent cohorts. Variant-level replication and attribution remain incomplete.	**Emerging**
**Other rare CNVs congenital hemivertebra / CS**	Lai 2021 analyzed 67 hemivertebra cases and 125 unaffected family controls using WES-derived CNV calling. Beyond known 16p11.2 deletions, heterogeneous candidate CNVs were reported at multiple loci. [42]	Locus-specific deletions or duplications with heterogeneous and often incomplete inheritance information.	qPCR confirmed selected CNVs; gene-level biological support was variable and frequently indirect.	CNV is an alteration class, not one disease relationship. Most individual loci lack independent replication and complete gene/mechanism attribution; WES has boundary and non-coding coverage limitations.	**Hypothesis-generating (exploratory category only)**
**Other non-coding regulatory variation CVM**	No replicated CVM-specific non-coding relationship was identified beyond the T-C-A component already evaluated within *TBX6* compound inheritance. [8]	Undefined locus-specific regulatory mechanisms; phase and target-gene attribution generally unresolved.	General WGS detection and non-coding interpretation methods are not disease-specific functional validation.	Do not infer pathogenicity from location or in-silico prediction alone. The *TBX6* T-C-A evidence is counted only in the canonical *TBX6* row.	**Hypothesis-generating (exploratory category only)**
***KAT6B* promoter methylation CS / Cobb-angle association**	Wu 2020 compared peripheral blood from 50 CS cases and 50 controls and reported an association with Cobb angle; a separate within-study tissue/cell cohort was used for expression and mechanism experiments. [59]	Postnatal molecular association; no inherited causal epigenetic mechanism or segregation model established.	Facet-joint tissue and chondrocyte experiments support an *EZH2*/H3K27me3-*KAT6B*-*RUNX2*/Wnt hypothesis.	Cross-sectional blood methylation cannot distinguish cause, modifier, cell-composition effect or consequence. No diagnostic or prognostic performance has been externally validated.	**Hypothesis-generating**
***TNS3* promoter methylation CS association**	Wu 2022 used two discordant monozygotic twin pairs, followed by 50-case/50-control blood validation and 20-case/20-control tissue-expression analysis. [58, 59]	Postnatal molecular association; no inherited causal epigenetic mechanism established.	Lower *TNS3* staining in articular-process cartilage provides supporting expression evidence.	No external replication. Hospital, recruitment dates, ethics identifier and 50+50 design overlap with Wu 2020, so sample overlap is possible and the cohorts are not added together.	**Hypothesis-generating**
**Tissue methylome differences hemivertebra versus spinal-process tissue**	Liu 2021 performed WGBS on hemivertebra and spinal-process specimens from four unrelated patients; no healthy same-tissue comparator or external replication was included. [60]	Within-patient cross-tissue molecular comparison, not a genetic segregation model.	Pathway enrichment and candidate differentially methylated regions were reported; transcriptome was not measured.	Different anatomical sites and cell composition may drive the signal. The study cannot establish causal, diagnostic or prognostic DMRs.	**Hypothesis-generating (exploratory category only)**

Specified relationship and phenotype	Human evidence and replication boundary	Inheritance and segregation	Functional or developmental evidence	Key limitations and clinical boundary	Tier
***ITPR2* ultra-rare variant burden**<br>**CVM risk**	Zhao 2024 recruited 873 CVM probands and 3,794 controls; burden analysis included 744 molecularly undiagnosed cases and 3,740 controls passing QC: 26 cases and 37 controls carried qualifying variants; reported OR 3.83 (95% CI 1.83-8.01), FDR 0.0088. External independent replication was not identified. [40]	Gene-level burden/susceptibility signal; no single universal inheritance model or automatic pathogenic classification for individual variants.	Developmental expression and human embryonic-spine single-nucleus enrichment support biological context, not variant-level causality.	One study; association does not establish every variant, penetrance, or clinical actionability.	**Emerging**
***TPO* ultra-rare variant burden**<br>**CVM risk**	Zhao 2024 (the same 744-case/3,740-control burden set): 12 cases and 12 controls with qualifying variants; reported OR 6.72 (95% CI 2.15-21.0), FDR 0.0274. External independent replication was not identified. [40]	Gene-level burden/susceptibility signal; established thyroid-disease relationships are not transferred to CVM variants.	Human embryonic-spine expression enrichment provides contextual support; CVM variant-level validation is incomplete.	One study and wide confidence interval. Burden evidence cannot classify each variant or establish an isolated-CVM diagnosis.	**Emerging**
***H6PD* ultra-rare variant burden**<br>**CVM risk**	Zhao 2024 (the same 744-case/3,740-control burden set): 11 cases and 26 controls with qualifying variants; reported OR 4.83 (95% CI 1.84-12.7), FDR 0.0338. External independent replication was not identified. [40]	Gene-level burden/susceptibility signal; reported case variants were predominantly heterozygous and heterogeneous.	Developmental expression and pathway context support plausibility; no complete CVM-specific variant-function series was presented.	One study; no penetrance estimate, single-variant diagnostic rule or proven axial mechanism.	**Emerging**
***SEC24B* ultra-rare variant burden**<br>**CVM risk**	Zhao 2024 (the same 744-case/3,740-control burden set): nine cases and 15 controls with qualifying variants; reported OR 5.77 (95% CI 1.55-21.4), FDR 0.0343. External independent replication was not identified. [40]	Gene-level burden/susceptibility signal; no general Mendelian CVM inheritance model established.	Trafficking and planar-cell-polarity biology provide plausibility; human embryonic-spine enrichment is contextual rather than variant-level validation.	One study and wide confidence interval. Directional developmental evidence does not establish each human variant or clinical use.	**Emerging**
***NADSYN1* biallelic variants**<br>**congenital NAD deficiency / VCRL spectrum**	Fourteen non-duplicate CNDD pedigrees: four in 2020 plus ten further families in 2024 [70, 71]. Vertebral anomalies occur in 3/5 individuals in 2020 and 10/12 with structural anomalies in 2024; this is not a count of fourteen vertebral-positive pedigrees.	Autosomal recessive syndromic relationship; complete biallelic genotypes and phenotype context are required.	Reduced protein abundance or NAD synthetase activity and NAD-pathway developmental models support the mechanism.	Established for the syndromic biallelic relationship, not for isolated CVM or for a single heterozygous VUS.	**Established**
***NADSYN1* heterozygous variants**<br>**sporadic CVM susceptibility**	Lin 2021 screened 424 CVM probands and reported eight heterozygous variants in nine unrelated patients, all with additional organ defects. Parental DNA was unavailable and the variants were classified as VUS. [44]	Heterozygous susceptibility or modifier model proposed; segregation and phase were unavailable.	Selected variants reduced protein abundance or enzymatic activity in COS-7 assays.	Heterozygous tolerance, absent parental data and lack of an independent cohort prevent transfer of the established biallelic syndrome relationship.	**Emerging**
DSTYK<br>**human CVM**	The cited Sun 2020 study was a zebrafish developmental study and did not provide an independent human CVM cohort establishing a *DSTYK* relationship. [45]	No human CVM-specific inheritance or segregation model established from the cited evidence.	*Dstyk* disruption produced congenital-scoliosis-like vertebral malformations in zebrafish through altered mTORC1/TFEB signaling.	Strong model relevance does not establish human disease validity or diagnostic utility.	**Hypothesis-generating**

Specified relationship and phenotype	Human evidence and replication boundary	Inheritance and segregation	Functional or developmental evidence	Key limitations and clinical boundary	Tier
***COL11A2* rare missense variants vertebral malformations / CS susceptibility**	Rebello 2023 identified two patients in an isolated-VM sequencing cohort and a third patient from a separate study. Two unrelated patients carried p.Arg130Trp; in one family the variant was inherited from a clinically unaffected mother. [46]	Proposed heterozygous susceptibility/haploinsufficiency relationship with incomplete penetrance; segregation is incomplete.	col11a2 loss-of-function zebrafish developed vertebral fusions; patient-variant constructs failed to rescue the model as effectively as wild type.	Only three human patients, limited parental data and an unaffected carrier. Functional rescue strengthens plausibility but does not establish full penetrance or clinical actionability.	**Emerging**
***FBN1* rare variants CVM / congenital scoliosis-specific relationship**	Lin 2020 analyzed 615 CS cases. One family had a segregating truncating variant; 574 sporadic cases versus 828 controls showed enrichment of predicted deleterious variants (OR 3.9; P=0.03); p.Leu871Phe recurred in two unrelated cases. [47]	Proposed heterozygous dominant/susceptibility relationship; evidence differs by allele and family.	p.Leu871Phe increased TGF-beta signaling in vitro.	Current evidence is limited and allele dependent. Established Marfan and other fibrillinopathy relationships do not validate every CVM candidate allele.	**Emerging**
***SOX9* p.Met469Val nonsyndromic CVM**	Wu 2019 reported the same rare heterozygous variant in three CVM patients without other systemic deformities. External human replication of the same narrow relationship was not identified in the current appraisal. [48]	Proposed heterozygous allele-specific relationship; complete family penetrance and segregation information remain limited.	Luciferase testing assessed altered transactivation; *SOX9* chondrogenic biology supports plausibility.	One small study. The established *SOX9*-campomelic-dysplasia relationship cannot be transferred to this selected allele or to isolated CVM generally.	**Emerging**

Abbreviations: ACMG/AMP, American College of Medical Genetics and Genomics/Association for Molecular Pathology; CNV, copy-number variant; CS, congenital scoliosis; CVM, congenital vertebral malformation; DMR, differentially methylated region; FDR, false-discovery rate; OFCS2, otofaciocervical syndrome type 2; OR, odds ratio; SCDO, spondylocostal dysostosis; VCRL, vertebral–cardiac–renal–limb; VUS, variant of uncertain significance; WES, whole-exome sequencing; WGBS, whole-genome bisulfite sequencing. Numbered citations correspond to the main-manuscript reference list. For rows 15, 16 and 19, Hypothesis-generating refers only to the exploratory observation category and does not assign gene–disease validity to every locus within it.

Table S2b. Condition-by-condition record for the 29 Table S2a entries

Read together with Table S2a and S5. Record numbers refer to appraisal entries, excluding the header. E1: Y, a direct human target-vertebral phenotype is documented; N, no qualifying human CVM evidence identified in the reviewed source set; A, aggregate observation category rather than a specific validity relationship. E2 describes independence and replication; E3 records genetic coherence and the principal unresolved condition. E4: NI, no material relationship-level contradiction identified in the sources appraised; B, an observation or uncertainty constrains a stronger model, as explained in S2a; -, a specific causal relationship was not established for this criterion. NI does not mean an exhaustive negative search or proof that no conflicting evidence exists. These descriptive codes are not points, variant classifications or a new validated scale. The existing tier labels are unchanged.

ID	Specified relationship	E1	E2 replication and source	E3 genetic coherence and unresolved evidence	E4	Existing tier
01	*TBX6* canonical compound genotype	Y	Discovery and additional series [8]; subsequent Japanese and international evidence [10,31]. Re-reports excluded.	Rare disruptive allele plus hypomorphic haplotype in trans; a coherent dosage model, not either component alone.	NI	Established
02	*TBX6* noncanonical biallelic missense	Y	Several pedigrees [28,61,62], but replication of one consistently specified allele-class mechanism is incomplete.	Allele pairing and functional effects differ. Normal transcription and abnormal localization are different readouts; one common mechanism is not established.	B	Emerging
03	16p11.2 gain including *TBX6*	Y	Four of seven selected carriers had CVMs involving the cervical spine [29]. Earlier duplication reports described spinal phenotypes, but their comparability with this specific cervical pattern remains uncertain.	Multigene gain and a dosage model are plausible; causal attribution to *TBX6* alone and segregation remain incomplete.	NI	Emerging
04	*DLL3* biallelic SCDO1	Y	Foundational families [14] followed by explicitly additional families [34]; not 13 independent mutation origins.	Compatible recessive disease and function-disrupting alleles support the shared relationship; not every allele was assayed.	NI	Established
05	*MESP2* biallelic SCDO2 and allelic STD	Y	Initial family [15] and subsequent, non-overlapping eligible pedigrees [63]. Single-allele family excluded.	Coherent recessive impaired-function spectrum. SCDO/STD subtype tendencies do not establish deterministic allele-to-anatomy rules.	NI	Established
06	*LFNG* biallelic SCDO3	Y	Initial report [12] plus later clinical reports [64,75]; duplicate case excluded. Functional reappraisal [65] is not a new family.	Recessive disease with relevant variant-function support. VUS-containing reports are descriptive cases, not automatic qualifying molecular diagnoses.	NI	Established
07	*HES7* biallelic SCDO4	Y	Separately reported families in [13,16,66]; extended branches in one kindred counted once.	Compatible recessive segregation and impaired repression/dimerization support the relationship; laterality is variably expressed.	NI	Established

ID	Specified relationship	E1	E2 replication and source	E3 genetic coherence and unresolved evidence	E4	Existing tier
08	*DLL1* p.Gly512Arg recessive vertebral relationship	Y	Two affected brothers in one family [39]; no qualifying independent family identified.	Compatible segregation and patient-sample readouts provide support; complete variant-mechanism validation is lacking.	NI	Emerging
09	*RIPPLY2* biallelic SCDO6	Y	Non-overlapping clinical families in [35,67] and subsequent reports [36,37]; repeated alleles alone are not patient duplication.	Compatible recessive genotypes and truncating-variant repression evidence [35]. A splice-region VUS is not upgraded by this relationship label.	NI	Established
10	*PAX1* biallelic OFCS2	Y	Initial syndrome report [55] and later families [56,77-80]; the re-reported Paganini pedigree in [77] is counted once.	Recessive syndrome evidence, including documented vertebral abnormalities [77]. Replication concerns OFCS2, not an isolated-CVM relationship or every pedigree being vertebral-positive.	NI	Established
11	*PAX1* candidate heterozygous CVM relationship	Y	One candidate report [69]; independent CVM-specific replication not identified.	Variants also occurred in unaffected mothers; no coherent replicated isolated-CVM inheritance model. Animal relevance is not human causal proof.	B	Hypothesis-generating
12	*PAX9* human CVM candidate	N	No qualifying independent human *PAX9*-CVM data identified in the reviewed source set.	Experimental *Pax1*/*Pax9* synergy [53,54] does not supply a human CVM genotype or segregation model. Tooth-agenesis validity is separate.	-	Hypothesis-generating
13	*VANGL1* CVM susceptibility	Y	One 782-patient study [23]. Multicenter recruitment and recurrent alleles within it are not a second independently ascertained study.	Rare hypomorphic/dominant-negative candidates plus cellular/model evidence; family data and variant-specific attribution are incomplete.	NI	Emerging
14	*VANGL2* CVM susceptibility	Y	Same study and shared 19-carrier set as *VANGL1* [23]; not independent replication between the two rows.	Model and cellular support for susceptibility, not a universally penetrant Mendelian CVM mechanism.	NI	Emerging
15	Other rare CNVs observation category	A	Not a single relationship. Heterogeneous loci in [42] require separate overlap and replication assessments.	qPCR confirmation establishes a copy-number finding, not the causal validity of every locus or affected gene.	-	Hypothesis-generating category
16	Other noncoding variation observation category	A	No one target locus/mechanism is graded. The supported T-C-A component remains in record 01 [8].	Computational regulatory annotation is not CVM-specific human segregation or functional causality.	-	Hypothesis-generating category

ID	Specified relationship	E1	E2 replication and source	E3 genetic coherence and unresolved evidence	E4	Existing tier
17	*KAT6B* methylation and CS/Cobb angle	Y	One program [59]; independent external replication not established.	Postnatal association and tissue/cell support do not establish developmental temporal order, inherited cause or validated prediction.	B	Hypothesis-generating
18	*TNS3* methylation and CS	Y	Twin and validation samples within one program [58]; possible overlap with [59] prevents independent pooling.	Postnatal methylation/expression findings; tissue and cell-composition confounding and causal direction remain unresolved.	B	Hypothesis-generating
19	Cross-tissue methylome observation category	A	Four-patient cross-tissue comparison [60]; not replicated validity for each DMR.	Different anatomical tissues and no healthy same-tissue comparator preclude causal attribution to individual methylation signals.	-	Hypothesis-generating category
20	*ITPR2* CVM ultra-rare burden	Y	One shared case-control analysis [40]; external replication not identified.	QC-passed burden association supports susceptibility; expression supplies context, not each variant's causal classification.	NI	Emerging
21	*TPO* CVM ultra-rare burden	Y	Same analysis as record 20 [40]; not a separate CVM cohort.	Gene-level association; CVM-specific variant mechanism and segregation are unresolved. Thyroid-disease validity is separate.	NI	Emerging
22	*H6PD* CVM ultra-rare burden	Y	Same analysis as record 20 [40]; no independent external replication identified.	Gene-level association with heterogeneous variants; a single Mendelian CVM mechanism and penetrance are not established.	NI	Emerging
23	*SEC24B* CVM ultra-rare burden	Y	Same analysis as record 20 [40]; no independent external replication identified.	Association plus trafficking/PCP plausibility; not a validated diagnostic rule for every human allele.	NI	Emerging
24	*NADSYN1* biallelic CNDD/VCRL spectrum	Y	Initial families [70] and ten further families [71]; syndrome pedigrees and vertebral-positive individuals remain separate counts.	Biallelic metabolic disease with reduced protein/enzyme activity; applicable to the syndrome, not one heterozygous VUS or isolated CVM.	NI	Established
25	*NADSYN1* heterozygous CVM susceptibility	Y	Nine patients in one series [44]; independent replication not identified.	Functional support for selected variants, but no parental DNA and VUS classifications. Heterozygous tolerance limits a dominant monogenic interpretation.	B	Emerging
26	*DSTYK* human CVM candidate	N	The cited study [45] is zebrafish work, not an independently ascertained human CVM series.	Model disruption supports developmental relevance but supplies no human CVM-specific inheritance evidence.	-	Hypothesis-generating

ID	Specified relationship	E1	E2 replication and source	E3 genetic coherence and unresolved evidence	E4	Existing tier
27	*COL11A2* CVM susceptibility	Y	Two discovery patients plus an additional unrelated p.Arg130Trp carrier from a separate study [46]; limited independent recurrence.	Variant rescue evidence supports plausibility, but only three patients and incomplete segregation. An unaffected transmitting mother limits a fully penetrant dominant model.	B	Emerging
28	*FBN1* CVM-specific susceptibility	Y	Familial segregation and case-control enrichment were reported in [47]. An earlier FBN1 pedigree with vertebral dysplasia is also discussed in that study; whether it supports the same congenital structural phenotype and variant mechanism remains uncertain.	Segregation and enrichment give human support; effects differ by allele and established Marfan relationships cannot validate all CVM alleles.	NI	Emerging
29	*SOX9* p.Met469Val nonsyndromic CVM	Y	Three carriers in one report [48]; independent replication of the same narrow relationship not identified.	Recurrent allele and reporter evidence support a candidate relationship; parental segregation and penetrance remain incomplete.	NI	Emerging
