# Supplementary material for: An Evidence-Tiered Developmental Framework for Congenital Vertebral Malformations: Spatiotemporal Thresholds in Genotype–Phenotype Interpretation
Source: Genes (Basel). 2026 Oct 21;17(10):1309. doi: 10.3390/genes17101309 (PMC13704003; doi:10.3390/genes17101309)
Supplement: Supplementary file 1 [file genes-17-01309-s001.zip › Supplementary TablesS1 and S3-S7.pdf]

These materials document the narrative review's retrieval limitations and relationship-specific evidence. Tables S1 and S3–S7 retain the manuscript citation numbering; Table S2 is supplied as a separate landscape file; its citations match the numbered references in the manuscript. Retrieval totals are not included-study counts. Original literature searching and screening were not independently duplicated; revision-stage tier assignments were independently reassessed by Qi Zhang.

Table S1a. Search provenance, eligibility and study selection

Search phase, database and date	Principal strategy and record counts	Eligibility and restrictions	Study selection and personnel
Original review, primary database: PubMed/MEDLINE. Final search: 31 May 2026.	Eight recovered strategies in Table S1c; partial original history. Original identified and included counts: not retained.	No language restriction. Eligibility boundaries are described below; other historical filters were not retained.	Xuhao Wang alone. Original deduplication, screening and exclusion counts: not retained. No independent duplicate screening.
Original review, supplementary searches: Web of Science, Embase, Scopus and Google Scholar. Final search: 31 May 2026.	Used to supplement the primary PubMed/MEDLINE search. Search terms: not retained. Identified and included counts: not retained.	No language restriction. Eligibility boundaries are described below; other historical filters were not retained.	Xuhao Wang alone. Original deduplication, screening and exclusion counts: not retained. No independent duplicate screening.
Revision source verification: PubMed-indexed records via Europe PMC API; 12 October 2026.	Eleven recorded topic queries; exact strings and returned/retrieved counts in Table S1b. Pool: 2207 unique identifiers; one broad query capped.	No language filter in the documented revision retrieval. Human genetic reports required congenital structural vertebral phenotypes; other literature informed mechanism or clinical context.	Targeted verification of original citations and selected primary reports by Xuhao Wang. No exhaustive eligibility screen or included-study count from the revision pool.

Original preparation. Xuhao Wang performed literature identification, relevance screening and initial evidence appraisal alone. PubMed/MEDLINE was the primary database; Web of Science, Embase, Scopus and Google Scholar were used for supplementary searches. The final search date was 31 May 2026 for all sources; no language restrictions were applied. Eight recovered PubMed/MEDLINE strategies are reproduced in Table S1c. Other original PubMed/MEDLINE queries and the search terms used in the supplementary databases were not retained. The original identified, deduplicated, screened, excluded and included record counts were also not retained. The recovered strategies therefore provide a partial search history; exhaustive coverage and the original selection flow cannot be substantiated. No independent duplicate screening or adjudication was performed.

Documented revision retrieval. PubMed-indexed records were retrieved through the Europe PMC API on 12 October 2026, using SRC:MED and the exact first-publication-date interval 1900-01-01 to 2026-10-12. No language filter was applied. The eleven exact queries and their hit and retrieval counts appear below. Records were de-duplicated by indexed record identifier, giving 2,207 unique records. This is a source-verification pool, not an included-study or systematically screened-study count.

Table S1b. Exact revision-stage PubMed-indexed retrieval strategies

Query	Exact strategy	Hits / retrieved	Complete retrieval of returned hits
Q1_genetic_architecture	(TITLE_ABS:"congenital vertebral" OR TITLE_ABS:"congenital scoliosis" OR TITLE_ABS:hemivertebra OR TITLE_ABS:"spondylocostal dysostosis") AND (TITLE_ABS:genetic* OR TITLE_ABS:genom* OR TITLE_ABS:inheritance) AND SRC:MED AND FIRST_PDATE:[1900-01-01 TO 2026-10-12]	242 / 242	Yes
Q2_segmentation	(TITLE_ABS:*DLL3* OR TITLE_ABS:*MESP2* OR TITLE_ABS:*LFNG* OR TITLE_ABS:*HES7* OR TITLE_ABS:*DLL1* OR TITLE_ABS:*RIPPLY2*) AND (TITLE_ABS:vertebr* OR TITLE_ABS:spondylocostal OR TITLE_ABS:scoliosis) AND SRC:MED AND FIRST_PDATE:[1900-01-01 TO 2026-10-12]	219 / 219	Yes
Q3_sclerotome	(TITLE_ABS:*PAX1* OR TITLE_ABS:*PAX9*) AND (TITLE_ABS:vertebr* OR TITLE_ABS:sclerotome OR TITLE_ABS:otofaciocervical OR TITLE_ABS:scoliosis) AND SRC:MED AND FIRST_PDATE:[1900-01-01 TO 2026-10-12]	142 / 142	Yes
Q4_planar_polarity	(TITLE_ABS:*VANGL1* OR TITLE_ABS:*VANGL2*) AND (TITLE_ABS:vertebr* OR TITLE_ABS:scoliosis) AND SRC:MED AND FIRST_PDATE:[1900-01-01 TO 2026-10-12]	92 / 92	Yes
Q5_clinical_testing	(TITLE_ABS:"congenital vertebral" OR TITLE_ABS:"congenital scoliosis" OR TITLE_ABS:hemivertebra OR TITLE_ABS:"spondylocostal dysostosis") AND (TITLE_ABS:exome OR TITLE_ABS:genome OR TITLE_ABS:microarray OR TITLE_ABS:"copy number" OR TITLE_ABS:prenatal) AND SRC:MED AND FIRST_PDATE:[1900-01-01 TO 2026-10-12]	125 / 125	Yes
Q6_three_dimensional	(TITLE_ABS:"congenital vertebral" OR TITLE_ABS:"congenital scoliosis" OR TITLE_ABS:hemivertebra OR TITLE_ABS:"spondylocostal dysostosis") AND (TITLE_ABS:"three dimensional" OR TITLE_ABS:"three-dimensional" OR TITLE_ABS:3D OR TITLE_ABS:biplanar OR TITLE_ABS:morphometr*) AND SRC:MED AND FIRST_PDATE:[1900-01-01 TO 2026-10-12]	90 / 90	Yes
Q7_heart_vertebral	(TITLE_ABS:vertebr* OR TITLE_ABS:hemivertebra OR TITLE_ABS:scoliosis) AND (TITLE_ABS:"congenital heart" OR TITLE_ABS:cardiac) AND (TITLE_ABS:genetic* OR TITLE_ABS:chromosom* OR TITLE_ABS:syndrome OR TITLE_ABS:cohort OR TITLE_ABS:"meta-analysis") AND SRC:MED AND FIRST_PDATE:[1900-01-01 TO 2026-10-12]	1596 / 1000	No; capped
Q8_environment_mosaicism	(TITLE_ABS:"congenital vertebral" OR TITLE_ABS:"congenital scoliosis" OR TITLE_ABS:hemivertebra OR TITLE_ABS:"spondylocostal dysostosis") AND (TITLE_ABS:hypoxia OR TITLE_ABS:maternal OR TITLE_ABS:diabetes OR TITLE_ABS:teratogen* OR TITLE_ABS:mosaic* OR TITLE_ABS:environment* OR TITLE_ABS:epigenetic*) AND SRC:MED AND FIRST_PDATE:[1900-01-01 TO 2026-10-12]	82 / 82	Yes
Q9_interpretation_guidelines	(TITLE_ABS:"gene-disease validity" OR TITLE_ABS:"interpretation of sequence variants" OR TITLE_ABS:"constitutional copy number variants" OR TITLE_ABS:"exome and genome sequencing for pediatric patients" OR TITLE_ABS:"sequencing for the diagnosis of fetal genetic disease") AND SRC:MED AND FIRST_PDATE:[1900-01-01 TO 2026-10-12]	166 / 166	Yes

Query	Exact strategy	Hits / retrieved	Complete retrieval of returned hits
Q10_recent_reviews	(TITLE_ABS:"congenital vertebral" OR TITLE_ABS:"congenital scoliosis" OR TITLE_ABS:hemivertebra OR TITLE_ABS:"spondylocostal dysostosis") AND (PUB_TYPE:review OR TITLE_ABS:review) AND SRC:MED AND FIRST_PDATE:[1900-01-01 TO 2026-10-12]	337 / 337	Yes
Q7b_heart_CVM_focus	(TITLE_ABS:"congenital vertebral" OR TITLE_ABS:"congenital scoliosis" OR TITLE_ABS:hemivertebra OR TITLE_ABS:"spondylocostal dysostosis") AND (TITLE_ABS:"congenital heart" OR TITLE_ABS:cardiac OR TITLE_ABS:dextrocardia) AND SRC:MED AND FIRST_PDATE:[1900-01-01 TO 2026-10-12]	90 / 90	Yes

Retrieval completeness. The broad vertebral–cardiac query returned 1,596 hits but retrieval was capped at 1,000. The focused Q7b query returned and retrieved 90 records. It does not recover or establish complete retrieval of the remaining broad-query hits. Hit counts describe the indexed database on the recorded search date and may change as records are updated.

Sources considered. Human reports were considered when they documented congenital structural vertebral abnormalities, relevant genetic findings, inheritance or segregation, associated multisystem phenotypes, diagnostic testing or clinical phenotyping. Experimental reports were considered when they informed an axial-development mechanism discussed in the review. Idiopathic or acquired scoliosis without congenital structural vertebral evidence was not used to establish CVM gene–disease validity. Reviews, disease summaries and professional guidance served as context or leads to primary reports, rather than independent human replication. Confirmed or possible reuse of families and cohorts was recorded and not treated as independent replication.

Source-checking process. Verification focused on original references and selected primary reports relevant to specific manuscript claims, using indexed metadata, abstracts and accessible full texts. Primary reports were prioritized for numerical, segregation and functional details. Inaccessible or unverifiable details were omitted or retained only at a directional level. A complete eligibility screen of the entire retrieval pool was not documented; no per-record include/exclude totals or reasons are supplied. No duplicate independent screening, adjudication or formal risk-of-bias assessment was performed. These logs describe revision-stage source verification rather than a reconstructed original systematic search.

Table S1c Recovered original PubMed MEDLINE search strategies

The following eight author-recovered strategies preserve the original PubMed operators, terms and field tags; whitespace has been standardized. The final original search date was 31 May 2026, with no language restriction. P1–P8 are reporting labels. Other original queries and per-query record counts were not retained. Broad clinical or automated-measurement retrieval supplies context and does not redefine human genetic eligibility.

ID and topic	Database and final original date	Exact recovered strategy
P1<br>Broad congenital vertebral and spinal malformation terms	PubMed/MEDLINE<br>31 May 2026<br>No language restriction	`("Scoliosis"[Mesh] AND (congenital[tiab] OR "vertebral malformation*"[tiab] OR "vertebral anomal*"[tiab] OR "segmentation defect*"[tiab])) OR "congenital scoliosis"[tiab] OR "congenital vertebral malformation*"[tiab] OR "congenital vertebral anomal*"[tiab] OR "congenital spinal deformit*"[tiab] OR "congenital spine deformit*"[tiab] OR "hemivertebra*"[tiab] OR "spondylocostal dysostosis"[tiab] OR "spondylothoracic dysostosis"[tiab] OR "Klippel-Feil syndrome"[tiab]`

ID and topic	Database and final original date	Exact recovered strategy
P2<br>Human genetics and genomic variation	PubMed/MEDLINE<br>31 May 2026<br>No language restriction	("congenital scoliosis"[tiab] OR "congenital vertebral malformation*"[tiab] OR "vertebral segmentation defect*"[tiab] OR "spondylocostal dysostosis"[tiab] OR "spondylothoracic dysostosis"[tiab] OR "Klippel-Feil syndrome"[tiab]) AND (genet*[tiab] OR genomic*[tiab] OR mutation*[tiab] OR variant*[tiab] OR "compound inheritance"[tiab] OR haploinsufficien*[tiab] OR "gene dosage"[tiab] OR "copy number variant*"[tiab] OR TBX6[tiab] OR PAX1[tiab] OR PAX9[tiab] OR DLL3[tiab] OR DLL1[tiab] OR MESP2[tiab] OR LFNG[tiab] OR HES7[tiab] OR RIPPLY2[tiab] OR VANGL1[tiab] OR VANGL2[tiab] OR NADSYN1[tiab] OR DSTYK[tiab] OR FBN1[tiab] OR SOX9[tiab] OR COL11A2[tiab])
P3<br>Vertebral development and segmentation mechanisms	PubMed/MEDLINE<br>31 May 2026<br>No language restriction	(somitogenesis[tiab] OR somite*[tiab] OR "segmentation clock"[tiab] OR "vertebral segmentation"[tiab] OR sclerotome[tiab] OR "vertebral column"[tiab] OR "axial skeleton"[tiab] OR "paraxial mesoderm"[tiab] OR "notochord morphogenesis"[tiab] OR somitoid*[tiab]) AND (develop*[tiab] OR pattern*[tiab] OR segment*[tiab] OR embryo*[tiab] OR molecular[tiab] OR mechanis*[tiab] OR organoid*[tiab] OR PAX1[tiab] OR PAX9[tiab] OR TBX6[tiab] OR MESP2[tiab] OR HES7[tiab] OR LFNG[tiab] OR RIPPLY2[tiab] OR NOTCH[tiab] OR FGF[tiab] OR WNT[tiab])
P4<br>Epigenetic and environmental mechanisms	PubMed/MEDLINE<br>31 May 2026<br>No language restriction	("congenital scoliosis"[tiab] OR "congenital vertebral malformation*"[tiab] OR "vertebral segmentation defect*"[tiab] OR "vertebral malformation*"[tiab] OR "axial skeletal defect*"[tiab]) AND (epigenet*[tiab] OR methylation[tiab] OR "DNA methylation"[tiab] OR "histone modification*"[tiab] OR transcriptom*[tiab] OR "gene-environment interaction*"[tiab] OR "environmental factor*"[tiab] OR "environmental stress*"[tiab] OR hypoxi*[tiab] OR metabol*[tiab] OR "NAD deficiency"[tiab])
P5<br>Associated anomalies and clinical epidemiology	PubMed/MEDLINE<br>31 May 2026<br>No language restriction	("congenital scoliosis"[tiab] OR "congenital vertebral malformation*"[tiab] OR "congenital vertebral anomal*"[tiab] OR hemivertebra*[tiab]) AND ("associated anomal*"[tiab] OR "concomitant anomal*"[tiab] OR "intraspinal anomal*"[tiab] OR "spinal cord anomal*"[tiab] OR "cardiac anomal*"[tiab] OR "renal anomal*"[tiab] OR "rib anomal*"[tiab] OR "organ system anomal*"[tiab] OR comorbid*[tiab] OR epidemiolog*[tiab] OR incidence[tiab] OR prevalence[tiab])
P6<br>Prenatal imaging and molecular diagnosis	PubMed/MEDLINE<br>31 May 2026<br>No language restriction	(fetal[tiab] OR prenatal[tiab] OR antenatal[tiab]) AND (hemivertebra*[tiab] OR "vertebral anomal*"[tiab] OR "vertebral abnormalit*"[tiab] OR "vertebral defect*"[tiab] OR "skeletal anomal*"[tiab] OR "skeletal abnormalit*"[tiab]) AND (diagnos*[tiab] OR screening[tiab] OR ultrasound[tiab] OR ultrasonograph*[tiab] OR MRI[tiab] OR "magnetic resonance imaging"[tiab] OR "exome sequencing"[tiab] OR "genome sequencing"[tiab] OR WES[tiab] OR WGS[tiab] OR sequenc*[tiab] OR outcome*[tiab])
P7<br>Treatment and management	PubMed/MEDLINE<br>31 May 2026<br>No language restriction	("congenital scoliosis"[tiab] OR "congenital spinal deformit*"[tiab] OR "congenital vertebral malformation*"[tiab] OR hemivertebra*[tiab]) AND (treatment[tiab] OR management[tiab] OR surgery[tiab] OR surgical[tiab] OR "hemivertebra resection"[tiab] OR "spinal fusion"[tiab] OR "in situ fusion"[tiab] OR "surgical outcome*"[tiab] OR "long-term outcome*"[tiab] OR "long-term follow-up"[tiab] OR rehabilitation[tiab])

ID and topic	Database and final original date	Exact recovered strategy
P8<br>Automated spinal measurement and artificial intelligence	PubMed/MEDLINE<br>31 May 2026<br>No language restriction	`(scoliosis[tiab] OR "spinal deformit*"[tiab] OR "vertebral deformit*"[tiab]) AND ("artificial intelligence"[tiab] OR "machine learning"[tiab] OR "deep learning"[tiab] OR "automated measurement"[tiab] OR "computer-aided diagnosis"[tiab] OR ("Cobb angle"[tiab] AND (automated[tiab] OR algorithm*[tiab])))`

Table S2 Detailed evidence appraisal supplied as a separate landscape file

The separate Table S2 evaluates 29 appraisal entries: 26 named relationships and three broad exploratory observation categories. Its six columns record the phenotype and mechanism, human evidence and replication boundary, inheritance and segregation, functional or developmental evidence, limitations and clinical boundary, and assigned tier. Xuhao Wang assigned the initial tiers and Qi Zhang independently reassessed all 29 against the criteria in use at that stage; all tier labels agreed. The expanded condition record in Table S2b documents these judgments and does not constitute a new independent reassessment. Missing or unreported penetrance, expressivity and segregation information is not treated as negative evidence.

Table S3 Context specific prenatal diagnostic yields

Study / assay	Tested population	Reported diagnoses	Interpretation boundary
Zhou 2022 chromosomal testing / CMA	Isolated fetal hemivertebra: 35 tested	4/35 (11.4%)	Selected tested subset of 109 total fetuses; not all isolated CVMs [128].
Zhou 2022 chromosomal testing / CMA	Non-isolated fetal hemivertebra: 23 tested	2/23 (8.7%)	No significant subgroup difference; denominator is tested fetuses, not whole cohort.
Zhou 2022 WES after normal CMA	Isolated fetal hemivertebra: 9 selected	1/9 (11.1%)	Very small selected subset; cannot establish a population yield range.
Zhou 2022 WES after normal CMA	Non-isolated fetal hemivertebra: 7 selected	2/7 (28.6%)	No significant subgroup difference; apparent numerical difference is not robust superiority.
Zhou 2022 WES total	Selected normal-CMA subset: 16	3/16 (18.75%; approximately 18.8%)	Calculated from full-text counts; abstract states 18.3%, which is arithmetically inconsistent.
Hu 2025 overall prenatal testing pathway [123]	Isolated fetal vertebral abnormalities: 33 fetuses	7/33 (21.2%)	Chromosomal analysis and selective WES; overall-pathway yield, not WES alone.
Hu 2025 overall prenatal testing pathway [123]	Non-isolated fetal vertebral abnormalities: 19 fetuses	3/19 (15.8%)	No significant subgroup difference was reported. These two groups account for 10/52 overall diagnoses; source-defined non-isolated findings are not all multisystem CVMs.

Study / assay	Tested population	Reported diagnoses	Interpretation boundary
Xie 2025 overall prenatal testing pathway [122]	Isolated fetal vertebral defects: 8 fetuses	2/8 (25.0%)	CMA with additional WES after negative CMA; overall-pathway yield, not WES alone.
Xie 2025 overall prenatal testing pathway [122]	Non-isolated fetal vertebral defects: 4 fetuses	3/4 (75.0%)	Very small subgroup; reported Fisher exact P = 0.141. The two groups account for 5/12 overall diagnoses; no established non-isolated yield advantage.
Broader fetal skeletal / structural studies	Different eligibility, assays and anomaly populations	Not pooled here	These are not isolated-versus-multisystem CVM-specific estimates [133, 134, 136].

The Zhou WES total is calculated from the published numerator and denominator: 3/16 = 18.75% (approximately 18.8%); the abstract reports 18.3%. Hu and Xie percentages describe overall prenatal testing pathways, not WES-only yields. Non-isolated retains each source definition and is not re-labelled as all multisystem CVMs. No universal isolated-versus-multisystem yield range is pooled from these selected studies. Non-significant subgroup comparisons do not establish equivalent yields.

Table S4 Comparison with prior reviews and models

Prior work	Scope and overlap	Present emphasis and limit
Szoszkiewicz 2024 [19]	Human molecular landscape, new loci and future directions	Relationship-specific appraisal with explicit clinical boundaries; does not replace the molecular catalogue.
Zhao 2025 [20]	Genetic and epigenetic etiology	Cause/modifier/consequence and variant-validity distinctions; no claim of first epigenetic synthesis.
Samarkhanova 2025 [21]	Congenital spinal deformity mechanisms/classification	Operational CVM definitions and disease-specific tier assignment.
Feng 2024 [23]	Primary PCP genetic, dosage and gene–environment threshold model	Direct antecedent; the proposed broader synthesis is not a new first threshold model.

Table S5 Operational evidence tier criteria

Tier	Human support	Inheritance and phenotype	Functional support	Interpretation limits
Established (E)	Direct human target-vertebral phenotype; at least one independent non-overlapping dataset beyond discovery; substantive genetic support; no material unresolved contradiction. In this review, each Established Mendelian relationship has 5–14 non-duplicate pedigrees (Table S7).	Specified inheritance and variant mechanism must cohere with phase, segregation and phenotype.	Relevant model, tissue, stage, allele and rescue evidence is assessed separately; it cannot replace human replication.	Relationship-level tier only; no automatic variant pathogenicity, full penetrance or genotype-only prognosis.

Tier	Human support	Inheritance and phenotype	Functional support	Interpretation limits
Emerging (M)	Direct human genetic support for the specified relationship, but one or more E conditions remain unmet or incompletely resolved; record each unresolved condition. Minimum: E1 met with germline genetic evidence plus at least one of (i) a statistically significant controlled association, (ii) co-segregation in at least one family or (iii) functional evidence specific to the patient alteration, such as an assay of the patient allele or a model reproducing the same dosage change.	Record unresolved phase, uninformative or discordant segregation, unaffected carriers and phenotype range.	Mechanistic support can strengthen plausibility but does not cure absent human replication.	Not a validated diagnostic or prognostic rule; material contradictions may narrow or withhold the claim.
Hypothesis generating (H)	Does not meet the Emerging minimum; evidence is mainly indirect or exploratory. Non-genetic molecular associations, such as accessible-tissue DNA methylation, cannot exceed this tier.	Inheritance, causal direction or phenotype specificity is not established.	Pathway, model, expression or methylation findings are contextual, not direct human causal proof.	Research hypothesis only. Materially contradicted claims may be left unassigned rather than given H.

Decision rule. Define the relationship by gene or locus, variant mechanism, inheritance and vertebral phenotype before assigning a tier. For E, all four conditions in the table are required; an independent replication dataset may be published in the same paper if it was separately recruited and does not overlap the discovery sample. Multiple families within one discovery series show recurrence but are not an external replication dataset. No universal affected-family or case-number threshold is imposed. Record cohort overlap, phase and segregation, relevant unaffected carriers, phenotype severity and distribution, and any ascertainment limits on penetrance. Missing evidence is distinguished from evidence against the claim. Functional observations are described by allele specificity, axial tissue or developmental stage, phenotype readout and rescue. Contradictions are assessed for the exact relationship and may lower, narrow or withhold assignment. For the three broad exploratory entries in Table S2, H denotes the evidence state of that observation category only and does not classify every gene or locus in the category. These author-defined tiers are not formal ClinGen scores, individual ACMG/AMP or CNV variant classes, or clinical utility claims.

Table S5 continued. Condition-level interpretation of the author-defined evidence framework

Code	Condition	Evidence needed	Handling of limits
E1	Direct human target phenotype	A cited human report documents a congenital structural vertebral defect in the specified relationship; scoliosis alone or an animal phenotype does not suffice. In syndromic rows, the target is the named syndrome with reported vertebral involvement, not isolated CVM or vertebral involvement in every family.	Record morphology and syndromic context separately. Missing imaging detail is missing evidence, not proof of a normal vertebra.
E2	Independent human replication	At least one separately ascertained non-overlapping family or cohort beyond discovery supports the same gene/locus, inheritance and mechanistic relationship. Two independent sources are a minimum, not a sufficient total evidence threshold.	Separately recruited discovery and additional samples may appear in one paper. Multiple families in one discovery series, center count, re-reports or functional reanalysis do not create external replication.
E3	Coherent genetic support	For Mendelian relationships, evaluate compatible rare alleles, genotype/phase, segregation and the specified mechanism across human observations. For susceptibility, evaluate recurrence or a controlled association with appropriate variant filters and analysis; this does not establish a Mendelian diagnosis.	A shared gene name, in-silico prediction, expression, an isolated VUS or model phenotype alone is insufficient. Functional results are recorded by allele, model, tissue/stage, comparator and rescue when reported; they cannot replace human replication.
E4	No material unresolved contradiction	Evaluate credible opposing genetic or functional observations for the exact proposed relationship and their study quality, phenotype definition, ancestry, allele class and sample overlap. Narrow, downgrade or withhold a claim when a material contradiction remains unresolved.	Unreported evidence is not counterevidence. Unaffected carriers constrain penetrance claims but do not automatically refute susceptibility. Different assays are not contradictory unless they test the same claim under comparable conditions.

Pooling and strength of genetic support. Alleles are combined only within a compatible gene/locus, inheritance, target phenotype spectrum and mechanistic class. Different credible impaired-function alleles may support one recessive disease relationship without being the same sequence change. A multigene duplication, a rare loss-of-function plus hypomorphic genotype and noncanonical missense/missense combinations are not interchangeable. For a candidate susceptibility relationship, recurrence, segregation or a controlled association can provide direct support while missing replication or uncertain mechanistic coherence prevents an Established label. Mere molecular detection or an animal phenotype is not sufficient direct human support.

Segregation, penetrance and expressivity. Record tested relatives, allele phase when relevant, affected and unaffected carriers and the phenotype definition supplied by the source. Do not label segregation informative when relatives were not tested. Fractions in selected clinical carriers are not population penetrance estimates; numerical penetrance is not inferred without a defined genotyped at-risk denominator and adequate phenotypic ascertainment. Variation in anatomical distribution or severity is expressivity, not automatically failed segregation.

Functional and opposing evidence. Distinguish assays of a patient allele from normal gene biology. Where reported, record the allele, wild-type or other comparator, model, developmental tissue/stage, relevant readout and rescue; a generic pathway match is contextual support. Unreported controls or rescue are not invented. Apparent opposing findings are compared for the same phenotype, allele class and experimental claim before being called contradictory. An unaffected carrier can constrain a fully penetrant model without excluding susceptibility. Credible material contradiction can narrow, lower or withhold the relationship claim. These records are qualitative and are not a validated point score or a formal risk-of-bias assessment.

Assessment process. Xuhao Wang performed the original literature search, relevance screening and initial evidence appraisal. During revision, Qi Zhang independently reassessed tier assignments for all 29 Table S2 appraisal entries using the criteria in use at that stage. Agreement was 29 of 29; there were no disagreements to resolve or adjudicate. Original searching and screening were not duplicated, and no formal inter-rater statistic was calculated. The condition-by-condition record added in the present revision documents the qualitative framework explicitly; it is not a further independent reassessment or a new reliability analysis.

Table S6. Developmental windows, testable mechanisms and human-evidence boundaries

Stage and relative developmental window	Cell/tissue population and molecular mechanism	Expected malformation pattern (hypothesis)	Potential quantitative experimental readout	Level of supporting human evidence and boundary
Paraxial specification; before successive somite budding (relative, not a patient-specific gestational week)	Paraxial mesoderm/PSM; *TBX6* dosage and lineage competence	Formation or segmentation defects may occur; neither pattern is uniquely predicted by *TBX6* dosage.	Dose–response of lineage allocation and the fraction of competent somite-like units; independently differentiated cultures.	Established for the specified *TBX6* compound-inheritance relationship [7, 8]; assignment of an embryonic window to an individual radiographic pattern is unproven.
Clock and boundary formation; repeated presomitic segmentation cycles	PSM; Notch/Wnt/FGF oscillation and *DLL3*/*LFNG*/*HES7*–*MESP2*/*RIPPLY2* boundary regulation	Multilevel vertebral–rib segmentation abnormalities are expected for certain biallelic SCDO relationships, but level and severity vary.	Clock period and coherence; proportion of regular somite boundaries; boundary position and spacing.	Established human evidence for specified SCDO gene–disease relationships [11, 12, 13]; human in vitro models measure segmentation-clock period, coherence and boundary formation [84, 85, 88], and one study reports an experimental YAP-dependent excitability threshold [88]. These are experimental readouts, not numerical thresholds for CVM risk in patients.
Sclerotome differentiation; after somite formation	Ventromedial somite/sclerotome; SHH–*PAX1*/*PAX9* identity program	Vertebral/disc identity or shape may be altered; a unique isolated-CVM signature is not established.	Proportion of correctly specified sclerotome cells; spatial domain and condensation-marker integrity.	Established for biallelic *PAX1*-related syndromic disease [55, 56]; isolated *PAX1*-CVM and human *PAX9*-CVM remain hypothesis-generating.
Resegmentation and chondrogenesis; later axial differentiation	Adjacent sclerotome domains; resegmentation, *SOX9* and extracellular-matrix organization	Altered vertebral/disc shape or relationships may result; morphology alone does not reveal causal stage.	Correctly resegmented-domain fraction, cartilage differentiation and quantitative matrix organization.	Developmental and model support [100, 102]; no general validated human stage-to-malformation rule.

Stage and relative developmental window	Cell/tissue population and molecular mechanism	Expected malformation pattern (hypothesis)	Potential quantitative experimental readout	Level of supporting human evidence and boundary
Tissue morphogenesis; overlapping axial-development intervals	PCP/notochord and mechanical environment; VANGL-dependent polarity and tissue geometry	Changes in vertebral distribution or shape may occur; there is no unique PCP radiographic signature.	Cell-polarity orientation, tissue geometry and notochord-structure metrics across dose/exposure conditions.	Emerging human *VANGL1*/*VANGL2* susceptibility associations [23]; dose/environment effects are experimental, not universal patient thresholds [23, 104].

Windows are relative developmental intervals, not unverified human gestational dates. In vitro differentiation days and mouse embryonic stages are model-specific and cannot be converted directly into human timing. Expected patterns are proposed relations, not deterministic molecular imaging diagnoses.

Table S7a Source-defined pedigree counts for Table 1b

Audit date: 14 October 2026. These counts cover the named reports below, not every published family worldwide. A family is one pedigree unit; case-only reports are treated as one proband/pedigree unit unless overlap is identified. GeneReviews and historical literature tables are used for cross-checking but are not extra family series. Counts do not establish variant pathogenicity, independence of mutation origins, penetrance or clinical prediction. Access limitations are identified for each report.

Gene / specified relationship	Retained units	Arithmetic	Interpretation boundary
TBX6	3	1 + 1 + 1 = 3	Missense/missense genotypes only; three different combinations, not three replications of one exact genotype.
DLL3	13	3 + 10 = 13	Thirteen pedigrees in the two foundational papers; later worldwide families are outside this subtotal.
MESP2	13	1 + (13 reported − 1 single-allele pedigree) = 13	Thirteen genotype-complete pedigrees; one single-allele family excluded. Founder pedigrees are not independent mutation origins.
LFNG	10	1 + 2 + 1 + 1 + 1 + 0 + 1 + 3 + 0 = 10	Ten reported pedigree/case units; some alleles are VUS. One re-report removed; functional re-use contributes zero.
HES7	5	1 + 1 + 3 = 5	Five reported pedigree units; the extended 2013 kindred is not split into additional units.
RIPPLY2	6	1 + 1 + 2 + 2 = 6	Six pedigrees; sisters/brothers share units. Variant-level splice confidence remains separate.
PAX1	7	1 + 1 + 1 + (3 reported − 1 duplicate) + 1 + 1 = 7	Seven syndrome pedigrees; one re-report removed. Not seven vertebral-positive or isolated-CVM pedigrees.
NADSYN1	14	4 + 10 = 14	Fourteen syndrome pedigrees; congenital malformations and vertebral abnormalities are not present in every individual.

Table S7b Primary report audit and duplicate decisions

Gene and primary report	Reported family or case details	Count	Duplicate or eligibility decision	Evidence location and access
TBX6<br>Lefebvre 2017 [61]<br>10.1111/cge.12918	Four *TBX6*-positive patients; one has two rare missense variants.	1	Retain the missense/missense SCD pedigree. Exclude two deletion/risk-haplotype cases and one deletion/missense case from this narrowly defined row.	DOI-verified indexed abstract; the full clinical paper was not openly accessible.
TBX6<br>Otomo 2019 [28]<br>10.1136/jmedgenet-2018-105920	One Japanese SCD patient with biallelic missense variants; other positive cases use different mechanisms.	1	Count the Japanese missense/missense pedigree once. Functional reassessment of previously published variants adds no family.	Indexed Results; the genotype is also described in the Discussion of Chen 2020.
TBX6<br>Chen 2020 [62]<br>10.1002/humu.23907	Egyptian-ancestry US case US-P2/TSRH-P1: homozygous p.Leu140Phe.	1	US-P2 and TSRH-P1 designate the same patient. Exclude the Chinese and Hispanic risk-haplotype cases. The Japanese case cited in the Discussion is not new here.	Full text: identification of functional variants, Table 1 and Discussion.
DLL3<br>Bulman 2000 [14]<br>10.1038/74307	Three autosomal recessive SCD families.	3	Founding pedigrees; count families, not the number of variants.	DOI-verified indexed abstract.
DLL3<br>Turnpenny 2003 [34]<br>10.1136/jmg.40.5.333	Ten additional families; twelve mutation findings.	10	The paper explicitly describes these as further families. Add ten to the three founding pedigrees; do not add twelve mutations.	Primary abstract/full-text archive abstract; older scanned article body is not represented as fully extracted.
MESP2<br>Whittock 2004 [15]<br>10.1086/421053	One Lebanese consanguineous pedigree with two affected siblings.	1	One pedigree with segregating homozygous 4-bp duplication. The additional mutation-negative screened patients are excluded.	Full text: clinical family, sequencing and Figure 1.
MESP2<br>Cornier 2008 [63]<br>10.1016/j.ajhg.2008.04.014	One Boston pedigree, ten Puerto Rican pedigrees in Table 2, and two additional pedigrees in Figure 3.	12	Thirteen pedigrees were described, but Table 2 family 10 (patient 000408-05) has only one identified allele and is excluded. Related cousins in Figure 3B remain one extended pedigree.	Full text: Boston discovery case; Table 2; paragraph on the unidentified second allele; Figure 3.
LFNG<br>Sparrow 2006 [12]<br>10.1086/498879	One founding Lebanese pedigree, p.Phe188Leu homozygous.	1	Count the original clinical pedigree once; later functional re-use adds no pedigree.	Full text; reconciled with Lecca 2023 Table 1.
LFNG<br>Lefebvre 2018 [72]<br>10.1136/jmedgenet-2017-104939	Two *LFNG*-positive cases with different biallelic genotypes.	2	Retain two separately reported case/pedigree units. Later literature-table repetition adds no cases. Some alleles remain VUS.	Indexed cohort Results; individual genotypes cross-checked in Lecca 2023 Table 1.

Gene and primary report	Reported family or case details	Count	Duplicate or eligibility decision	Evidence location and access
LFNG<br>Maddirevula 2018 [73]<br>10.1038/gim.2018.50	One Arab newborn with homozygous p.Thr254Met.	1	Lecca 2023 Table 1 combines this report and Shamseldin 2021 in one patient row; retain a single unit rather than two.	Indexed primary cohort; case-level attribution and overlap reconciled from Lecca 2023 Table 1.
LFNG<br>Takeda 2018 [41]<br>10.1002/mgg3.466	Japanese 16-year-old male, p.Leu156Arg/p.Arg286Trp.	1	This is distinct from the infant reported by Otomo 2019: age and both alleles differ. Count once.	Full text: *LFNG*-positive case, Figure 1, and enzyme assays.
LFNG<br>Otomo 2019 [75]<br>10.1038/s10038-018-0548-2	Japanese infant, p.Lys124Asnfs*21/p.Asp201Asn.	1	A distinct *LFNG* case, not the *TBX6* paper numbered 28 and not the Takeda 2018 *LFNG* case.	DOI-verified indexed primary abstract; Lecca 2023 Table 1 gives age and allele pairing.
LFNG<br>Shamseldin 2021 [74]<br>10.1186/s13073-021-00973-0	Re-report of the Arab p.Thr254Met case.	0	Do not add a second pedigree: both papers occupy a single clinical row in Lecca 2023 Table 1.	Primary full text/index metadata; overlap explicitly reconciled in Lecca 2023 Table 1.
LFNG<br>Schuhmann 2021 [76]<br>10.1111/cge.13952	One 17-year-old with homozygous p.Thr149Ile.	1	Different genotype and clinical record from the preceding patients; retain one reported case/pedigree unit. Variant-level uncertainty remains.	The full two-page primary letter documents a 17-year-old female patient with homozygous *LFNG* p.(Thr149Ile), vertebral segmentation defects and rib anomalies.
LFNG<br>Lecca 2023 [64]<br>10.1111/cge.14336	Three new pedigrees: families 1, 2 and 3.	3	Add only the three new pedigrees. The seven earlier individuals summarized in Table 1 are already counted above. Several new alleles were classified as VUS.	Full author-institution PDF: Table 1, genetic findings and Figure 1A.
LFNG<br>Wengryn 2024 [65]<br>10.1096/fj.202302651rr	Functional analysis of nine previously associated missense variants.	0	No new clinical pedigree is enumerated in the accessible abstract. The functional reappraisal therefore contributes no additional verified pedigree to this audit.	DOI-verified indexed abstract.
HES7<br>Sparrow 2008 [13]<br>10.1093/hmg/ddn272	One founding autosomal recessive pedigree.	1	Count the autozygosity-mapped founding pedigree once.	DOI-verified indexed primary abstract.
HES7<br>Sparrow 2010 [16]<br>10.1038/ejhg.2009.241	One southern European pedigree with two affected offspring.	1	The two siblings are one pedigree. Do not count the two variant alleles as two families.	Full text: clinical Results and Figure 1.
HES7<br>Sparrow 2013 [66]<br>10.1002/ajmg.a.36073	One extended kindred plus two unrelated families; seven affected individuals in total.	3	Retain three pedigree units. Related branches in the extended kindred are not additional independent pedigrees. The same variant across the two unrelated families does not establish a separate mutation origin.	DOI-verified indexed abstract; family count and seven individuals cross-checked in GeneReviews Figure 3.

Gene and primary report	Reported family or case details	Count	Duplicate or eligibility decision	Evidence location and access
RIPPLY2<br>McInerney 2015 [35]<br>10.1093/hmg/ddu534	Two brothers with compound-heterozygous variants.	1	Two affected brothers form one segregating pedigree.	DOI-verified indexed clinical abstract.
RIPPLY2<br>Karaca 2015 [67]<br>10.1002/ajmg.a.37263	One consanguineous pedigree, homozygous c.299delT.	1	Retain the new KFS/heterotaxy pedigree; do not add its comparison with the previously reported *RIPPLY2* brothers.	Full-text archive and indexed primary abstract.
RIPPLY2<br>Serey 2020 [36]<br>10.1002/ajmg.a.61549	Three new patients from two unrelated families.	2	Add two new pedigrees, not three patient records or the historical cases mentioned in the Introduction.	DOI-verified indexed primary clinical abstract.
RIPPLY2<br>Wegler 2021 [37]<br>10.1111/cge.13916	Two sisters and one boy from two further families.	2	Sisters form one pedigree; the boy forms a second. Shared c.238A>T/c.240-4T>G alleles do not make these patients re-reports of the 2015 brothers. The splice allele remained VUS in this paper.	Full clinical PDF, Results, family descriptions and indexed abstract.
PAX1<br>Pohl 2013 [55]<br>10.1007/s00439-013-1337-9	One Turkish consanguineous pedigree with four affected members.	1	Count the original p.Gly166Val pedigree once, not four families.	DOI-verified indexed report; family details reconciled in Elbagoury 2023 Table 2.
PAX1<br>Paganini 2017 [78]<br>10.1111/cge.13085	One Moroccan pedigree with two affected children, p.Cys368*.	1	Count once; these are P2/P3 later reassessed by Yamazaki 2020.	Indexed primary abstract; explicit re-report statement in Yamazaki 2020 Results and Discussion.
PAX1<br>Patil 2018 [79]<br>10.1002/ajmg.a.38659	One new Indian pedigree with homozygous small insertion.	1	The report explicitly identifies a third OFCS2 family; retain one pedigree.	DOI-verified indexed abstract; Elbagoury 2023 Table 2.
PAX1<br>Yamazaki 2020 [77]<br>10.1126/sciimmunol.aax1036	Six studied patients from three families: P1, P2/P3 and P4–P6.	2	Exclude the P2/P3 pedigree already reported by Paganini 2017. Add the German P1 and Saudi P4–P6 pedigrees only.	Full text: Figure 1, Results and explicit duplicate statement in Discussion.
PAX1<br>Sherlaw 2022 [80]<br>10.1016/j.ejmg.2022.104523	One new female proband in an Iraqi-origin consanguineous pedigree.	1	Retain one new p.Ser168Alafs*4 pedigree; the previous five families are historical context.	DOI-verified indexed abstract; variant and ancestry cross-checked in Elbagoury 2023 Table 2.
PAX1<br>Elbagoury 2023 [56]<br>10.1007/s12031-023-02170-7	One new Egyptian pedigree, p.Gly405Argfs*51.	1	Add the new pedigree only. Table 2 re-summarizes six earlier pedigrees, which must not be added again.	Full text: patient report, Tables 1–2 and Discussion.
NADSYN1<br>Szot 2020 [70]<br>10.1016/j.ajhg.2019.12.006	Five individuals from four unrelated syndrome pedigrees.	4	Two siblings in family 1 count once. This is a CNDD pedigree count: vertebral anomalies were reported in three of five individuals, from two families.	Full text: Figure 1, Table 1 and clinical description.

Gene and primary report	Reported family or case details	Count	Duplicate or eligibility decision	Evidence location and access
NADSYN1<br>Szot 2024 [71]<br>10.1172/jci174824	Thirteen further individuals from ten unrelated syndrome pedigrees.	10	The paper explicitly identifies further families. Family 9 includes a biallelic sibling without congenital malformations; neither sibling is an extra family. Vertebral anomalies occur in ten of twelve individuals with structural anomalies.	Full text: Introduction, Results, Figure 1 and Table 1.

References

1. Sebaaly, A.; Daher, M.; Salameh, B.; Ghoul, A.; George, S.; Roukoz, S. Congenital scoliosis: a narrative review and proposal of a treatment algorithm. *EFORT Open Rev.* **2022**, *7*, 318–327. https://doi.org/10.1530/eor-21-0121.

2. Giampietro, P.F.; Raggio, C.L.; Blank, R.D.; McCarty, C.; Broeckel, U.; Pickart, M.A. Clinical, genetic and environmental factors associated with congenital vertebral malformations. *Mol. Syndromol.* **2013**, *4*, 94–105. https://doi.org/10.1159/000345329.

3. Arlet, V.; Odent, T.; Aebi, M. Congenital scoliosis. *Eur. Spine J.* **2003**, *12*, 456–463. https://doi.org/10.1007/s00586-003-0555-6.

4. Wu, N.; Liu, L.; Zhang, Y.; Wang, L.; Wang, S.; Zhao, S.; Li, G.; Yang, Y.; Lin, G.; Shen, J.; et al. Retrospective Analysis of Associated Anomalies in 636 Patients with Operatively Treated Congenital Scoliosis. *J. Bone Jt. Surg. Am.* **2023**, *105*, 537–548. https://doi.org/10.2106/jbjs.22.00277.

5. Sevencan, A.; Misir, A.; Ucpunar, H.; Balioglu, M.B.; Gur, V.; Akinci, S. The Incidence and Interrelationship of Concomitant Anomalies in Congenital Scoliosis. *Turk. Neurosurg.* **2019**, *29*, 404–408. https://doi.org/10.5137/1019-5149.jtn.24429-18.2.

6. Peng, Z.; Zhang, H.; Wang, S.; Zhang, J. Advances in the diagnosis and treatment of congenital scoliosis. *Eur. J. Med. Res.* **2025**, *30*, 683. https://doi.org/10.1186/s40001-025-02943-3.

7. Yang, N.; Wu, N.; Zhang, L.; Zhao, Y.; Liu, J.; Liang, X.; Ren, X.; Li, W.; Chen, W.; Dong, S.; et al. TBX6 compound inheritance leads to congenital vertebral malformations in humans and mice. *Hum. Mol. Genet.* **2019**, *28*, 539–547. https://doi.org/10.1093/hmg/ddy358.

8. Wu, N.; Ming, X.; Xiao, J.; Wu, Z.; Chen, X.; Shinawi, M.; Shen, Y.; Yu, G.; Liu, J.; Xie, H.; et al. TBX6 null variants and a common hypomorphic allele in congenital scoliosis. *N. Engl. J. Med.* **2015**, *372*, 341–350. https://doi.org/10.1056/nejmoa1406829.

9. Feng, X.; Cheung, J.P.Y.; Je, J.S.H.; Cheung, P.W.H.; Chen, S.; Yue, M.; Wang, N.; Choi, V.N.T.; Yang, X.; Song, Y.-Q.; et al. Genetic variants of TBX6 and TBXT identified in patients with congenital scoliosis in Southern China. *J. Orthop. Res.* **2021**, *39*, 971–988. https://doi.org/10.1002/jor.24805.

10. Takeda, K.; Kou, I.; Kawakami, N.; Iida, A.; Nakajima, M.; Ogura, Y.; Imagawa, E.; Miyake, N.; Matsumoto, N.; Yasuhiko, Y.; et al. Compound Heterozygosity for Null Mutations and a Common Hypomorphic Risk Haplotype in TBX6 Causes Congenital Scoliosis. *Hum. Mutat.* **2017**, *38*, 317–323. https://doi.org/10.1002/humu.23168.

11. Turnpenny, P.D.; Sloman, M.; Dunwoodie, S. Spondylocostal Dysostosis, Autosomal Recessive. In *GeneReviews*®; University of Washington, Seattle: Seattle, WA, USA, 2023. Available online: https://www.ncbi.nlm.nih.gov/books/NBK8828/ (accessed on 12 October 2026).

12. Sparrow, D.B.; Chapman, G.; Wouters, M.A.; Whittock, N.V.; Ellard, S.; Fatkin, D.; Turnpenny, P.D.; Kusumi, K.; Sillence, D.; Dunwoodie, S.L. Mutation of the LUNATIC FRINGE gene in humans causes spondylocostal dysostosis with a severe vertebral phenotype. *Am. J. Hum. Genet.* **2006**, *78*, 28–37. https://doi.org/10.1086/498879.

13. Sparrow, D.B.; Guillén-Navarro, E.; Fatkin, D.; Dunwoodie, S.L. Mutation of Hairy-and-Enhancer-of-Split-7 in humans causes spondylocostal dysostosis. *Hum. Mol. Genet.* **2008**, *17*, 3761–3766. https://doi.org/10.1093/hmg/ddn272.

14. Bulman, M.P.; Kusumi, K.; Frayling, T.M.; McKeown, C.; Garrett, C.; Lander, E.S.; Krumlauf, R.; Hattersley, A.T.; Ellard, S.; Turnpenny, P.D. Mutations in the human delta homologue, DLL3, cause axial skeletal defects in spondylocostal dysostosis. *Nat. Genet.* **2000**, *24*, 438–441. https://doi.org/10.1038/74307.

15. Whittock, N.V.; Sparrow, D.B.; Wouters, M.A.; Sillence, D.; Ellard, S.; Dunwoodie, S.L.; Turnpenny, P.D. Mutated MESP2 causes spondylocostal dysostosis in humans. *Am. J. Hum. Genet.* **2004**, *74*, 1249–1254. https://doi.org/10.1086/421053.

16. Sparrow, D.B.; Sillence, D.; Wouters, M.A.; Turnpenny, P.D.; Dunwoodie, S.L. Two novel missense mutations in HAIRY-AND-ENHANCER-OF-SPLIT-7 in a family with spondylocostal dysostosis. *Eur. J. Hum. Genet.* **2010**, *18*, 674–679. https://doi.org/10.1038/ejhg.2009.241.

17. Scaal, M. Early development of the vertebral column. *Semin. Cell Dev. Biol.* **2016**, *49*, 83–91. https://doi.org/10.1016/j.semcdb.2015.11.003.

18. Miao, Y.; Pourquié, O. Cellular and molecular control of vertebrate somitogenesis. *Nat. Rev. Mol. Cell Biol.* **2024**, *25*, 517–533. https://doi.org/10.1038/s41580-024-00709-z.

19. Szoszkiewicz, A.; Bukowska-Olech, E.; Jamsheer, A. Molecular landscape of congenital vertebral malformations: recent discoveries and future directions. *Orphanet J. Rare Dis.* **2024**, *19*, 32. https://doi.org/10.1186/s13023-024-03040-0.

20. Zhao, R.; Zhao, J.-R.; Xue, X.; Ma, D. Deciphering the etiology of congenital scoliosis: A genetic and epigenetic perspective. *World J. Orthop.* **2025**, *16*, 104853. https://doi.org/10.5312/wjo.v16.i6.104853.

21. Samarkhanova, D.; Zhabagin, M.; Nadirov, N. Reviewing the Genetic and Molecular Foundations of Congenital Spinal Deformities: Implications for Classification and Diagnosis. *J. Clin. Med.* **2025**, *14*, 1113. https://doi.org/10.3390/jcm14041113.

22. Feng, S.; Du, Y.; Choi, S.W.; Cheung, K.S.C.; Cheung, J.P.Y.; Su, Z. The role of genetics and molecular mechanisms in early onset scoliosis. *J. Clin. Orthop. Trauma* **2026**, *73*, 103340. https://doi.org/10.1016/j.jcot.2026.103340.

23. Feng, X.; Ye, Y.; Zhang, J.; Zhang, Y.; Zhao, S.; Mak, J.C.W.; Otomo, N.; Zhao, Z.; Niu, Y.; Yonezawa, Y.; et al. Core planar cell polarity genes VANGL1 and VANGL2 in predisposition to congenital vertebral malformations. *Proc. Natl. Acad. Sci. USA* **2024**, *121*, e2310283121. https://doi.org/10.1073/pnas.2310283121.

24. Sparrow, D.B.; Chapman, G.; Smith, A.J.; Mattar, M.Z.; Major, J.A.; O'Reilly, V.C.; Saga, Y.; Zackai, E.H.; Dormans, J.P.; Alman, B.A.; et al. A mechanism for gene-environment interaction in the etiology of congenital scoliosis. *Cell* **2012**, *149*, 295–306. https://doi.org/10.1016/j.cell.2012.02.054.

25. Strande, N.T.; Riggs, E.R.; Buchanan, A.H.; Ceyhan-Birsoy, O.; DiStefano, M.; Dwight, S.S.; Goldstein, J.; Ghosh, R.; Seifert, B.A.; Sneddon, T.P.; et al. Evaluating the Clinical Validity of Gene-Disease Associations: An Evidence-Based Framework Developed by the Clinical Genome Resource. *Am. J. Hum. Genet.* **2017**, *100*, 895–906. https://doi.org/10.1016/j.ajhg.2017.04.015.

26. Richards, S.; Aziz, N.; Bale, S.; Bick, D.; Das, S.; Gastier-Foster, J.; Grody, W.W.; Hegde, M.; Lyon, E.; Spector, E.; et al. Standards and guidelines for the interpretation of sequence variants: a joint consensus recommendation of the American College of Medical Genetics and Genomics and the Association for Molecular Pathology. *Genet. Med.* **2015**, *17*, 405–424. https://doi.org/10.1038/gim.2015.30.

27. Riggs, E.R.; Andersen, E.F.; Cherry, A.M.; Kantarci, S.; Kearney, H.; Patel, A.; Raca, G.; Ritter, D.I.; South, S.T.; Thorland, E.C.; et al. Technical standards for the interpretation and reporting of constitutional copy-number variants: a joint consensus recommendation of the American College of Medical Genetics and Genomics (ACMG) and the Clinical Genome Resource (ClinGen). *Genet. Med.* **2020**, *22*, 245–257. https://doi.org/10.1038/s41436-019-0686-8.

28. Otomo, N.; Takeda, K.; Kawai, S.; Kou, I.; Guo, L.; Osawa, M.; Alev, C.; Kawakami, N.; Miyake, N.; Matsumoto, N.; et al. Bi-allelic loss of function variants of TBX6 causes a spectrum of malformation of spine and rib including congenital scoliosis and spondylocostal dysostosis. *J. Med. Genet.* **2019**, *56*, 622–628. https://doi.org/10.1136/jmedgenet-2018-105920.

29. Ren, X.; Yang, N.; Wu, N.; Xu, X.; Chen, W.; Zhang, L.; Li, Y.; Du, R.-Q.; Dong, S.; Zhao, S.; et al. Increased TBX6 gene dosages induce congenital cervical vertebral malformations in humans and mice. *J. Med. Genet.* **2020**, *57*, 371–379. https://doi.org/10.1136/jmedgenet-2019-106333.

30. Chen, W.; Liu, J.; Yuan, D.; Zuo, Y.; Liu, Z.; Liu, S.; Zhu, Q.; Qiu, G.; Huang, S.; Giampietro, P.F.; et al. Progress and perspective of TBX6 gene in congenital vertebral malformations. *Oncotarget* **2016**, *7*, 57430–57441. https://doi.org/10.18632/oncotarget.10619.

31. Liu, J.; Wu, N.; Deciphering Disorders Involving Scoliosis and COmorbidities (DISCO) study; Yang, N.; Takeda, K.; Chen, W.; Li, W.; Du, R.; Liu, S.; Zhou, Y.; et al. TBX6-associated congenital scoliosis (TACS) as a clinically distinguishable subtype of congenital scoliosis: further evidence supporting the compound inheritance and TBX6 gene dosage model. *Genet. Med.* **2019**, *21*, 1548–1558. https://doi.org/10.1038/s41436-018-0377-x.

32. Chen, Z.; Yan, Z.; Yu, C.; Liu, J.; Zhang, Y.; Zhao, S.; Lin, J.; Zhang, Y.; Wang, L.; Lin, M.; et al. Cost-effectiveness analysis of using the TBX6-associated congenital scoliosis risk score (TACScore) in genetic diagnosis of congenital scoliosis. *Orphanet J. Rare Dis.* **2020**, *15*, 250. https://doi.org/10.1186/s13023-020-01537-y.

33. Lin, G.; Yang, Y.; Chen, Z.; Zhao, S.; Niu, Y.; Du, Y.; Zhao, Y.; Wang, S.; Wu, N.; Zhang, J. Preliminary study assessing the long-term surgical outcomes of TBX6-associated congenital scoliosis (TACS) patients using the propensity score matching method: exploring the clinical implications of genetic discoveries in congenital scoliosis. *Orphanet J. Rare Dis.* **2025**, *20*, 32. https://doi.org/10.1186/s13023-024-03471-9.

34. Turnpenny, P.D.; Whittock, N.; Duncan, J.; Dunwoodie, S.; Kusumi, K.; Ellard, S. Novel mutations in DLL3, a somitogenesis gene encoding a ligand for the Notch signalling pathway, cause a consistent pattern of abnormal vertebral segmentation in spondylocostal dysostosis. *J. Med. Genet.* **2003**, *40*, 333–339. https://doi.org/10.1136/jmg.40.5.333.

35. McInerney-Leo, A.M.; Sparrow, D.B.; Harris, J.E.; Gardiner, B.B.; Marshall, M.S.; O'Reilly, V.C.; Shi, H.; Brown, M.A.; Leo, P.J.; Zankl, A.; et al. Compound heterozygous mutations in RIPPLY2 associated with vertebral segmentation defects. *Hum. Mol. Genet.* **2015**, *24*, 1234–1242. https://doi.org/10.1093/hmg/ddu534.

36. Serey-Gaut, M.; Scala, M.; Reversade, B.; Ruaud, L.; Cabrol, C.; Musacchia, F.; Torella, A.; Accogli, A.; Escande-Beillard, N.; Langlais, J.; et al. Congenital posterior cervical spine malformation due to biallelic c.240-4T>G RIPPLY2 variant: A discrete entity. *Am. J. Med. Genet. A* **2020**, *182*, 1466–1472. https://doi.org/10.1002/ajmg.a.61549.

37. Wegler, M.; Roth, C.; Schumann, E.; Kogan, J.; Totten, E.; Guillen Sacoto, M.J.; Abou Jamra, R.; Hornemann, F. Congenital cervical spine malformation due to bi-allelic RIPPLY2 variants in spondylocostal dysostosis type 6. *Clin. Genet.* **2021**, *99*, 565–571. https://doi.org/10.1111/cge.13916.

38. Fischer-Zirnsak, B.; Segebrecht, L.; Schubach, M.; Charles, P.; Alderman, E.; Brown, K.; Cadieux-Dion, M.; Cartwright, T.; Chen, Y.; Costin, C.; et al. Haploinsufficiency of the Notch Ligand DLL1 Causes Variable Neurodevelopmental Disorders. *Am. J. Hum. Genet.* **2019**, *105*, 631–639. https://doi.org/10.1016/j.ajhg.2019.07.002.

39. Barhoumi, T.; Nashabat, M.; Alghanem, B.; Alhallaj, A.; Boudjelal, M.; Umair, M.; Alarifi, S.; Alfares, A.; Al Mohrij, S.A.; Alfadhel, M. Delta Like-1 Gene Mutation: A Novel Cause of Congenital Vertebral Malformation. *Front. Genet.* **2019**, *10*, 534. https://doi.org/10.3389/fgene.2019.00534.

40. Zhao, S.; Zhao, H.; Zhao, L.; Cheng, X.; Zheng, Z.; Wu, M.; Wen, W.; Wang, S.; Zhou, Z.; Xie, H.; et al. Unraveling the genetic architecture of congenital vertebral malformation with reference to the developing spine. *Nat. Commun.* **2024**, *15*, 1125. https://doi.org/10.1038/s41467-024-45442-5.

41. Takeda, K.; Kou, I.; Mizumoto, S.; Yamada, S.; Kawakami, N.; Nakajima, M.; Otomo, N.; Ogura, Y.; Miyake, N.; Matsumoto, N.; et al. Screening of known disease genes in congenital scoliosis. *Mol. Genet. Genom. Med.* **2018**, *6*, 966–974. https://doi.org/10.1002/mgg3.466.

42. Lai, W.; Feng, X.; Yue, M.; Cheung, P.W.H.; Choi, V.N.T.; Song, Y.-Q.; Luk, K.D.K.; Cheung, J.P.Y.; Gao, B. Identification of Copy Number Variants in a Southern Chinese Cohort of Patients with Congenital Scoliosis. *Genes* **2021**, *12*, 1213. https://doi.org/10.3390/genes12081213.

43. McDonald-McGinn, D.M.; Hain, H.S.; Emanuel, B.S.; Zackai, E.H. 22q11.2 Deletion Syndrome. In *GeneReviews®*; University of Washington, Seattle: Seattle, WA, USA, 2025. Available online: https://www.ncbi.nlm.nih.gov/books/NBK1523/ (accessed on 12 October 2026).

44. Lin, J.; Zhao, L.; Zhao, S.; Li, S.; Zhao, Z.; Chen, Z.; Zheng, Z.; Shao, J.; Niu, Y.; Li, X.; et al. Disruptive NADSYN1 Variants Implicated in Congenital Vertebral Malformations. *Genes* **2021**, *12*, 1615. https://doi.org/10.3390/genes12101615.

45. Sun, X.; Zhou, Y.; Zhang, R.; Wang, Z.; Xu, M.; Zhang, D.; Huang, J.; Luo, F.; Li, F.; Ni, Z.; et al. Dstyk mutation leads to congenital scoliosis-like vertebral malformations in zebrafish via dysregulated mTORC1/TFEB pathway. *Nat. Commun.* **2020**, *11*, 479. https://doi.org/10.1038/s41467-019-14169-z.

46. Rebello, D.; Wohler, E.; Erfani, V.; Li, G.; Aguilera, A.N.; Santiago-Cornier, A.; Zhao, S.; Hwang, S.W.; Steiner, R.D.; Zhang, T.J.; et al. COL11A2 as a candidate gene for vertebral malformations and congenital scoliosis. *Hum. Mol. Genet.* **2023**, *32*, 2913–2928. https://doi.org/10.1093/hmg/ddad117.

47. Lin, M.; Zhao, S.; Liu, G.; Huang, Y.; Yu, C.; Zhao, Y.; Wang, L.; Zhang, Y.; Yan, Z.; Wang, S.; et al. Identification of novel FBN1 variations implicated in congenital scoliosis. *J. Hum. Genet.* **2020**, *65*, 221–230. https://doi.org/10.1038/s10038-019-0698-x.

48. Wu, N.; Wang, L.; Hu, J.; Zhao, S.; Liu, B.; Li, Y.; Du, H.; Zhang, Y.; Li, X.; Yan, Z.; et al. A Recurrent Rare SOX9 Variant (M469V) is Associated with Congenital Vertebral Malformations. *Curr. Gene Ther.* **2019**, *19*, 242–247. https://doi.org/10.2174/1566523219666190924120307.

49. Yang, Y.; Zhao, S.; Zhang, Y.; Wang, S.; Shao, J.; Liu, B.; Li, Y.; Yan, Z.; Niu, Y.; Li, X.; et al. Mutational burden and potential oligogenic model of TBX6-mediated genes in congenital scoliosis. *Mol. Genet. Genom. Med.* **2020**, *8*, e1453. https://doi.org/10.1002/mgg3.1453.

50. Schipper, M.; Posthuma, D. Demystifying non-coding GWAS variants: an overview of computational tools and methods. *Hum. Mol. Genet.* **2022**, *31*, R73-R83. https://doi.org/10.1093/hmg/ddac198.

51. Wallin, J.; Wilting, J.; Koseki, H.; Fritsch, R.; Christ, B.; Balling, R. The role of Pax-1 in axial skeleton development. *Development* **1994**, *120*, 1109–1121. https://doi.org/10.1242/dev.120.5.1109.

52. Wilm, B.; Dahl, E.; Peters, H.; Balling, R.; Imai, K. Targeted disruption of Pax1 defines its null phenotype and proves haploinsufficiency. *Proc. Natl. Acad. Sci. USA* **1998**, *95*, 8692–8697. https://doi.org/10.1073/pnas.95.15.8692.

53. Peters, H.; Wilm, B.; Sakai, N.; Imai, K.; Maas, R.; Balling, R. Pax1 and Pax9 synergistically regulate vertebral column development. *Development* **1999**, *126*, 5399–5408. https://doi.org/10.1242/dev.126.23.5399.

54. Sivakamasundari, V.; Kraus, P.; Sun, W.; Hu, X.; Lim, S.L.; Prabhakar, S.; Lufkin, T. A developmental transcriptomic analysis of Pax1 and Pax9 in embryonic intervertebral disc development. *Biol. Open* **2017**, *6*, 187–199. https://doi.org/10.1242/bio.023218.

55. Pohl, E.; Aykut, A.; Beleggia, F.; Karaca, E.; Durmaz, B.; Keupp, K.; Arslan, E.; Palamar, M.; Yigit, G.; Özkinay, F.; et al. A hypofunctional PAX1 mutation causes autosomal recessively inherited otofaciocervical syndrome. *Hum. Genet.* **2013**, *132*, 1311–1320. https://doi.org/10.1007/s00439-013-1337-9.

56. Elbagoury, N.M.; Abdel-Aleem, A.F.; Sharaf-Eldin, W.E.; Ashaat, E.A.; Esswai, M.L. A Novel Truncating Mutation in PAX1 Gene Causes Otofaciocervical Syndrome Without Immunodeficiency. *J. Mol. Neurosci.* **2023**, *73*, 976–982. https://doi.org/10.1007/s12031-023-02170-7.

57. Ushiki, A.; Sheng, R.R.; Zhang, Y.; Zhao, J.; Nobuhara, M.; Murray, E.; Ruan, X.; Rios, J.J.; Wise, C.A.; Ahituv, N. Deletion of Pax1 scoliosis-associated regulatory elements leads to a female-biased tail abnormality. *Cell Rep.* **2024**, *43*, 113907. https://doi.org/10.1016/j.celrep.2024.113907.

58. Wu, Y.; Zhang, H.-Q.; Tang, M.; Guo, C.; Liu, S.; Li, J.; Wang, Y.; Xiao, L.; Yang, G. Abnormal TNS3 gene methylation in patients with congenital scoliosis. *BMC Musculoskelet. Disord.* **2022**, *23*, 797. https://doi.org/10.1186/s12891-022-05730-x.

59. Wu, Y.; Zhang, H.; Tang, M.; Guo, C.; Deng, A.; Li, J.; Wang, Y.; Xiao, L.; Yang, G. High methylation of lysine acetyltransferase 6B is associated with the Cobb angle in patients with congenital scoliosis. *J. Transl. Med.* **2020**, *18*, 210. https://doi.org/10.1186/s12967-020-02367-z.

60. Liu, G.; Zhao, H.; Yan, Z.; Zhao, S.; Niu, Y.; Li, X.; Wang, S.; Yang, Y.; Liu, S.; Zhang, T.J.; et al. Whole-genome methylation analysis reveals novel epigenetic perturbations of congenital scoliosis. *Mol. Ther. Nucleic Acids* **2021**, *23*, 1281–1287. https://doi.org/10.1016/j.omtn.2021.02.002.

61. Lefebvre, M.; Duffourd, Y.; Jouan, T.; Poe, C.; Jean-Marçais, N.; Verloes, A.; St-Onge, J.; Riviere, J.-B.; Petit, F.; Pierquin, G.; et al. Autosomal recessive variations of TBX6, from congenital scoliosis to spondylocostal dysostosis. *Clin. Genet.* **2017**, *91*, 908–912. https://doi.org/10.1111/cge.12918.

62. Chen, W.; Lin, J.; Wang, L.; Li, X.; Zhao, S.; Liu, J.; Akdemir, Z.C.; Zhao, Y.; Du, R.; Ye, Y.; et al. TBX6 missense variants expand the mutational spectrum in a non-Mendelian inheritance disease. *Hum. Mutat.* **2020**, *41*, 182–195. https://doi.org/10.1002/humu.23907.

63. Cornier, A.S.; Staehling-Hampton, K.; Delventhal, K.M.; Saga, Y.; Caubet, J.-F.; Sasaki, N.; Ellard, S.; Young, E.; Ramirez, N.; Carlo, S.E.; et al. Mutations in the MESP2 gene cause spondylothoracic dysostosis/Jarcho-Levin syndrome. *Am. J. Hum. Genet.* **2008**, *82*, 1334–1341. https://doi.org/10.1016/j.ajhg.2008.04.014.

64. Lecca, M.; Bedeschi, M.F.; Izzi, C.; Dordoni, C.; Rinaldi, B.; Peluso, F.; Caraffi, S.G.; Prefumo, F.; Signorelli, M.; Zanzucchi, M.; et al. Identification of bi-allelic LFNG variants in three patients and further clinical and molecular refinement of spondylocostal dysostosis 3. *Clin. Genet.* **2023**, *104*, 230–237. https://doi.org/10.1111/cge.14336.

65. Wengryn, P.; Fenrich, F.; Silveira, K.d.C.; Oborn, C.; Mizumoto, S.; Beke, A.; Soltys, C.-L.; Yamada, S.; Kannu, P. Integrative analysis of Lunatic Fringe variants associated with spondylocostal dysostosis type-III. *FASEB J.* **2024**, *38*, e23753. https://doi.org/10.1096/fj.202302651rr.

66. Sparrow, D.B.; Faqeih, E.A.; Sallout, B.; Alswaid, A.; Ababneh, F.; Al-Sayed, M.; Rukban, H.; Eyaid, W.M.; Kageyama, R.; Ellard, S.; et al. Mutation of HES7 in a large extended family with spondylocostal dysostosis and dextrocardia with situs inversus. *Am. J. Med. Genet. A* **2013**, *161A*, 2244–2249. https://doi.org/10.1002/ajmg.a.36073.

67. Karaca, E.; Yuregir, O.O.; Bozdogan, S.T.; Aslan, H.; Pehlivan, D.; Jhangiani, S.N.; Akdemir, Z.C.; Gambin, T.; Bayram, Y.; Atik, M.M.; et al. Rare variants in the notch signaling pathway describe a novel type of autosomal recessive Klippel-Feil syndrome. *Am. J. Med. Genet. A* **2015**, *167A*, 2795–2799. https://doi.org/10.1002/ajmg.a.37263.

68. Zhao, W.; Oginuma, M.; Ajima, R.; Kiso, M.; Okubo, A.; Saga, Y. Ripply2 recruits proteasome complex for Tbx6 degradation to define segment border during murine somitogenesis. *eLife* **2018**, *7*, e33068. https://doi.org/10.7554/elife.33068.

69. Giampietro, P.F.; Raggio, C.L.; Reynolds, C.E.; Shukla, S.K.; McPherson, E.; Ghebranious, N.; Jacobsen, F.S.; Kumar, V.; Faciszewski, T.; Pauli, R.M.; et al. An analysis of PAX1 in the development of vertebral malformations. *Clin. Genet.* **2005**, *68*, 448–453. https://doi.org/10.1111/j.1399-0004.2005.00520.x.

70. Szot, J.O.; Campagnolo, C.; Cao, Y.; Iyer, K.R.; Cuny, H.; Drysdale, T.; Flores-Daboub, J.A.; Bi, W.; Westerfield, L.; Liu, P.; et al. Bi-allelic Mutations in NADSYN1 Cause Multiple Organ Defects and Expand the Genotypic Spectrum of Congenital NAD Deficiency Disorders. *Am. J. Hum. Genet.* **2020**, *106*, 129–136. https://doi.org/10.1016/j.ajhg.2019.12.006.

71. Szot, J.O.; Cuny, H.; Martin, E.M.M.A.; Sheng, D.Z.; Iyer, K.; Portelli, S.; Nguyen, V.; Gereis, J.M.; Alankarage, D.; Chitayat, D.; et al. A metabolic signature for NADSYN1-dependent congenital NAD deficiency disorder. *J. Clin. Investig.* **2024**, *134*, e174824. https://doi.org/10.1172/jci174824.

72. Lefebvre, M.; Dieux-Coeslier, A.; Baujat, G.; Schaefer, E.; Judith, S.-O.; Bazin, A.; Pinson, L.; Attie-Bitach, T.; Baumann, C.; Fradin, M.; et al. Diagnostic strategy in segmentation defect of the vertebrae: a retrospective study of 73 patients. *J. Med. Genet.* **2018**, *55*, 422–429. https://doi.org/10.1136/jmedgenet-2017-104939.

73. Maddirevula, S.; Alsahli, S.; Alhabeeb, L.; Patel, N.; Alzahrani, F.; Shamseldin, H.E.; Anazi, S.; Ewida, N.; Alsaif, H.S.; Mohamed, J.Y.; et al. Expanding the phenome and variome of skeletal dysplasia. *Genet. Med.* **2018**, *20*, 1609–1616. https://doi.org/10.1038/gim.2018.50.

74. Shamseldin, H.E.; AlAbdi, L.; Maddirevula, S.; Alsaif, H.S.; Alzahrani, F.; Ewida, N.; Hashem, M.; Abdulwahab, F.; Abuyousef, O.; Kuwahara, H.; et al. Lethal variants in humans: lessons learned from a large molecular autopsy cohort. *Genome Med.* **2021**, *13*, 161. https://doi.org/10.1186/s13073-021-00973-0.

75. Otomo, N.; Mizumoto, S.; Lu, H.-F.; Takeda, K.; Campos-Xavier, B.; Mittaz-Crettol, L.; Guo, L.; Takikawa, K.; Nakamura, M.; Yamada, S.; et al. Identification of novel LFNG mutations in spondylocostal dysostosis. *J. Hum. Genet.* **2019**, *64*, 261–264. https://doi.org/10.1038/s10038-018-0548-2.

76. Schuhmann, S.; Koller, H.; Sticht, H.; Kraus, C.; Krumbiegel, M.; Uebe, S.; Ekici, A.B.; Reis, A.; Thiel, C.T. Clinical and molecular delineation of spondylocostal dysostosis type 3. *Clin. Genet.* **2021**, *99*, 851–852. https://doi.org/10.1111/cge.13952.

77. Yamazaki, Y.; Urrutia, R.; Franco, L.M.; Giliani, S.; Zhang, K.; Alazami, A.M.; Dobbs, A.K.; Masneri, S.; Joshi, A.; Otaizo-Carrasquero, F.; et al. PAX1 is essential for development and function of the human thymus. *Sci. Immunol.* **2020**, *5*, eaax1036. https://doi.org/10.1126/sciimmunol.aax1036.

78. Paganini, I.; Sestini, R.; Capone, G.L.; Putignano, A.L.; Contini, E.; Giotti, I.; Gensini, F.; Marozza, A.; Barilaro, A.; Porfirio, B.; et al. A novel PAX1 null homozygous mutation in autosomal recessive otofaciocervical syndrome associated with severe combined immunodeficiency. *Clin. Genet.* **2017**, *92*, 664–668. https://doi.org/10.1111/cge.13085.

79. Patil, S.J.; Das Bhowmik, A.; Bhat, V.; Satidevi Vineeth, V.; Vasudevamurthy, R.; Dalal, A. Autosomal recessive otofaciocervical syndrome type 2 with novel homozygous small insertion in PAX1 gene. *Am. J. Med. Genet. A* **2018**, *176*, 1200–1206. https://doi.org/10.1002/ajmg.a.38659.

80. Sherlaw-Sturrock, C.; Austin, T.; Baptista, J.; Gilmour, K.; Naik, S. Dysmorphism and immunodeficiency - One of the differential diagnoses is PAX1 related otofaciocervical syndrome type 2. *Eur. J. Med. Genet.* **2022**, *65*, 104523. https://doi.org/10.1016/j.ejmg.2022.104523.

81. Wahi, K.; Bochter, M.S.; Cole, S.E. The many roles of Notch signaling during vertebrate somitogenesis. *Semin. Cell Dev. Biol.* **2016**, *49*, 68–75. https://doi.org/10.1016/j.semcdb.2014.11.010.

82. Pourquié, O. Vertebrate segmentation: from cyclic gene networks to scoliosis. *Cell* **2011**, *145*, 650–663. https://doi.org/10.1016/j.cell.2011.05.011.

83. Aulehla, A.; Herrmann, B.G. Segmentation in vertebrates: clock and gradient finally joined. *Genes Dev.* **2004**, *18*, 2060–2067. https://doi.org/10.1101/gad.1217404.

84. Yamanaka, Y.; Hamidi, S.; Yoshioka-Kobayashi, K.; Munira, S.; Sunadome, K.; Zhang, Y.; Kurokawa, Y.; Ericsson, R.; Mieda, A.; Thompson, J.L.; et al. Reconstituting human somitogenesis in vitro. *Nature* **2023**, *614*, 509–520. https://doi.org/10.1038/s41586-022-05649-2.

85. Miao, Y.; Djeffal, Y.; De Simone, A.; Zhu, K.; Lee, J.G.; Lu, Z.; Silberfeld, A.; Rao, J.; Tarazona, O.A.; Mongera, A.; et al. Reconstruction and deconstruction of human somitogenesis in vitro. *Nature* **2023**, *614*, 500–508. https://doi.org/10.1038/s41586-022-05655-4.

86. Sanaki-Matsumiya, M.; Matsuda, M.; Gritti, N.; Nakaki, F.; Sharpe, J.; Trivedi, V.; Ebisuya, M. Periodic formation of epithelial somites from human pluripotent stem cells. *Nat. Commun.* **2022**, *13*, 2325. https://doi.org/10.1038/s41467-022-29967-1.

87. Budjan, C.; Liu, S.; Ranga, A.; Gayen, S.; Pourquié, O.; Hormoz, S. Paraxial mesoderm organoids model development of human somites. *eLife* **2022**, *11*, e68925. https://doi.org/10.7554/elife.68925.

88. Diaz-Cuadros, M.; Wagner, D.E.; Budjan, C.; Hubaud, A.; Tarazona, O.A.; Donelly, S.; Michaut, A.; Al Tanoury, Z.; Yoshioka-Kobayashi, K.; Niino, Y.; et al. In vitro characterization of the human segmentation clock. *Nature* **2020**, *580*, 113–118. https://doi.org/10.1038/s41586-019-1885-9.

89. Qin, Y.; Huang, X.; Cai, Z.; Cai, B.; He, J.; Yao, Y.; Zhou, C.; Kuang, J.; Yang, Y.; Chen, H.; et al. Regeneration of the human segmentation clock in somitoids in vitro. *EMBO J.* **2022**, *41*, e110928. https://doi.org/10.15252/embj.2022110928.

90. Nelemans, B.K.A.; Schmitz, M.; Tahir, H.; Merks, R.M.H.; Smit, T.H. Somite Division and New Boundary Formation by Mechanical Strain. *iScience* **2020**, *23*, 100976. https://doi.org/10.1016/j.isci.2020.100976.

91. Gomes de Almeida, P.; Rifes, P.; Martins-Jesus, A.P.; Pinheiro, G.G.; Andrade, R.P.; Thorsteinsdóttir, S. Cell-Fibronectin Interactions and Actomyosin Contractility Regulate the Segmentation Clock and Spatio-Temporal Somite Cleft Formation during Chick Embryo Somitogenesis. *Cells* **2022**, *11*, 2003. https://doi.org/10.3390/cells11132003.

92. Naganathan, S.R.; Oates, A.C. Patterning and mechanics of somite boundaries in zebrafish embryos. *Semin. Cell Dev. Biol.* **2020**, *107*, 170–178. https://doi.org/10.1016/j.semcdb.2020.04.014.

93. Chandel, A.S.; Stocker, M.; Özbudak, E.M. The Role of Fibroblast Growth Factor Signaling in Somitogenesis. *DNA Cell Biol.* **2023**, *42*, 580–584. https://doi.org/10.1089/dna.2023.0226.

94. Anderson, M.J.; Magidson, V.; Kageyama, R.; Lewandoski, M. Fgf4 maintains Hes7 levels critical for normal somite segmentation clock function. *eLife* **2020**, *9*, e55608. https://doi.org/10.7554/elife.55608.

95. Bhavna, R. Segmentation clock dynamics is strongly synchronized in the forming somite. *Dev. Biol.* **2020**, *460*, 55–69. https://doi.org/10.1016/j.ydbio.2019.03.008.

96. Williams, D.R.; Shifley, E.T.; Lather, J.D.; Cole, S.E. Posterior skeletal development and the segmentation clock period are sensitive to Lfng dosage during somitogenesis. *Dev. Biol.* **2014**, *388*, 159–169. https://doi.org/10.1016/j.ydbio.2014.02.006.

97. Keseroglu, K.; Zinani, O.Q.H.; Keskin, S.; Seawall, H.; Alpay, E.E.; Özbudak, E.M. Stochastic gene expression and environmental stressors trigger variable somite segmentation phenotypes. *Nat. Commun.* **2023**, *14*, 6497. https://doi.org/10.1038/s41467-023-42220-7.

98. Nóbrega, A.; Maia-Fernandes, A.C.; Andrade, R.P. Altered Cogs of the Clock: Insights into the Embryonic Etiology of Spondylocostal Dysostosis. *J. Dev. Biol.* **2021**, *9*, 5. https://doi.org/10.3390/jdb9010005.

99. Ebensperger, C.; Wilting, J.; Brand-Saberi, B.; Mizutani, Y.; Christ, B.; Balling, R.; Koseki, H. Pax-1, a regulator of sclerotome development is induced by notochord and floor plate signals in avian embryos. *Anat. Embryol.* **1995**, *191*, 297–310. https://doi.org/10.1007/bf00534682.

100. Ward, L.; Evans, S.E.; Stern, C.D. A resegmentation-shift model for vertebral patterning. *J. Anat.* **2017**, *230*, 290–296. https://doi.org/10.1111/joa.12540.

101. Keynes, R. Patterning spinal nerves and vertebral bones. *J. Anat.* **2018**, *232*, 534–539. https://doi.org/10.1111/joa.12714.

102. Draga, M.; Scaal, M. Building a vertebra: Development of the amniote sclerotome. *J. Morphol.* **2024**, *285*, e21665. https://doi.org/10.1002/jmor.21665.

103. Ueda, H.; Iimura, T.; Inami, S.; Moridaira, H.; Yazawa, T.; Seo, Y.; Taneichi, H. Histology and chronological magnetic resonance images of congenital spinal deformity: An experimental study in mice model. *BMC Musculoskelet. Disord.* **2024**, *25*, 334. https://doi.org/10.1186/s12891-024-07460-8.

104. Wang, M.; Zhao, S.; Shi, C.; Guyot, M.-C.; Liao, M.; Tauer, J.T.; Willie, B.M.; Cobetto, N.; Aubin, C.-É.; Küster-Schöck, E.; et al. Planar cell polarity zebrafish models of congenital scoliosis reveal underlying defects in notochord morphogenesis. *Development* **2024**, *151*, dev202829. https://doi.org/10.1242/dev.202829.

105. Dudakovic, A.; Camilleri, E.T.; Xu, F.; Riester, S.M.; McGee-Lawrence, M.E.; Bradley, E.W.; Paradise, C.R.; Lewallen, E.A.; Thaler, R.; Deyle, D.R.; et al. Epigenetic Control of Skeletal Development by the Histone Methyltransferase Ezh2. *J. Biol. Chem.* **2015**, *290*, 27604–27617. https://doi.org/10.1074/jbc.m115.672345.

106. Roidl, D.; Hacker, C. Histone methylation during neural development. *Cell Tissue Res.* **2014**, *356*, 539–552. https://doi.org/10.1007/s00441-014-1842-8.

107. Rai, S.; Leydier, L.; Sharma, S.; Katwala, J.; Sahu, A. A quest for genetic causes underlying signaling pathways associated with neural tube defects. *Front. Pediatr.* **2023**, *11*, 1126209. https://doi.org/10.3389/fped.2023.1126209.

108. Mohanty, S.P.; Pai Kanhangad, M.; Narayana Kurup, J.K.; Saiffudeen, S. Vertebral, intraspinal and other organ anomalies in congenital scoliosis. *Eur. Spine J.* **2020**, *29*, 2449–2456. https://doi.org/10.1007/s00586-020-06450-3.

109. Wang, X.; Yu, Y.; Yang, N.; Xia, L. Incidence of intraspinal abnormalities in congenital scoliosis: a systematic review and meta-analysis. *J. Orthop. Surg. Res.* **2020**, *15*, 485. https://doi.org/10.1186/s13018-020-02015-8.

110. Ghandhari, H.; Tari, H.V.; Ameri, E.; Safari, M.B.; Fouladi, D.F. Vertebral, rib, and intraspinal anomalies in congenital scoliosis: a study on 202 Caucasians. *Eur. Spine J.* **2015**, *24*, 1510–1521. https://doi.org/10.1007/s00586-015-3833-1.

111. Furdock, R.; Brouillet, K.; Luhmann, S.J. Organ System Anomalies Associated With Congenital Scoliosis: A Retrospective Study of 305 Patients. *J. Pediatr. Orthop.* **2019**, *39*, e190–e194. https://doi.org/10.1097/bpo.0000000000001279.

112. Lin, G.; Chai, X.; Wang, S.; Yang, Y.; Shen, J.; Zhang, J. Cross-sectional analysis and trend of vertebral and associated anomalies in Chinese congenital scoliosis population: a retrospective study of one thousand, two hundred and eighty nine surgical cases from 2010 to 2019. *Int. Orthop.* **2021**, *45*, 2049–2059. https://doi.org/10.1007/s00264-021-05061-x.

113. Lin, G.; Chai, X.; Wang, S.; Yang, Y.; Su, Z.; Du, Y.; Xu, X.; Ye, X.; Shen, J.; Zhang, J. Cross-sectional analysis of associated anomalies and vertebral anomaly location in 1289 surgical congenital scoliosis. *Eur. Spine J.* **2021**, *30*, 3577–3584. https://doi.org/10.1007/s00586-021-06898-x.

114. Wang, S.; Zhao, Y.; Du, Y.; Zhang, J.; Yu, B. The incidence and interrelationship of hemivertebra and concomitant cardiac abnormalities in congenital scoliosis. *BMC Musculoskelet. Disord.* **2023**, *24*, 621. https://doi.org/10.1186/s12891-023-06727-w.

115. Bas, J.L.; Pérez, S.; Rubio, P.; Mariscal, G.; Bonilla, F.; Bovea, M.; Bas, P.; Bas, T. Incidence of cardiac anomalies in congenital vertebral deformity: systematic review and meta-analysis of 2910 patients. *Eur. Spine J.* **2023**, *32*, 2967–2974. https://doi.org/10.1007/s00586-023-07817-y.

116. Spinner, N.B.; Loomes, K.M.; Krantz, I.D.; Gilbert, M.A. Alagille Syndrome. In *GeneReviews*®; University of Washington, Seattle: Seattle, WA, USA, 2024. Available online: https://www.ncbi.nlm.nih.gov/books/NBK1273/ (accessed on 12 October 2026).

117. Humbert, L.; Steffen, J.-S.; Vialle, R.; Dubousset, J.; Vital, J.-M.; Skalli, W. 3D analysis of congenital scoliosis due to hemivertebra using biplanar radiography. *Eur. Spine J.* **2013**, *22*, 379–386. https://doi.org/10.1007/s00586-012-2539-x.

118. Avellanet, M.; Sanchez-Raya, J.; Maccarone, M.C.; Dionyssiotis, Y. The role of artificial intelligence in advancing scoliosis care: a rapid review of current evidence and future opportunities. *Front. Med.* **2026**, *13*, 1774697. https://doi.org/10.3389/fmed.2026.1774697.

119. Li, H.; Qian, C.; Yan, W.; Fu, D.; Zheng, Y.; Zhang, Z.; Meng, J.; Wang, D. Use of Artificial Intelligence in Cobb Angle Measurement for Scoliosis: Retrospective Reliability and Accuracy Study of a Mobile App. *J. Med. Internet Res.* **2024**, *26*, e50631. https://doi.org/10.2196/50631.

120. Jawed, A.M.; Zhang, L.; Zhang, Z.; Liu, Q.; Ahmed, W.; Wang, H. Artificial intelligence and machine learning in spine care: Advancing precision diagnosis, treatment, and rehabilitation. *World J. Orthop.* **2025**, *16*, 107064. https://doi.org/10.5312/wjo.v16.i8.107064.

121. Lam, C.; Tasong, J.; Bulut, H.; Udall, A.; Sukhbaatar, T.; Hoang, G.; Koye, A.; Ahn, J.; Ghazi, F.; Loader, D.; et al. Artificial intelligence in early onset scoliosis: a scoping review. *Spine Deform.* **2026**, *14*, 389–397. https://doi.org/10.1007/s43390-025-01208-7.

122. Xie, W.; Zhou, L.; Hu, A.; Chen, J.; Pang, J.; Xi, H.; Luo, Y.; Hu, J.; Yang, S.; Gao, X.; et al. Ultrasound and genetic findings in a case series of fetuses presenting vertebral defects. *BMC Pregnancy Childbirth* **2025**, *25*, 858. https://doi.org/10.1186/s12884-025-07920-6.

123. Hu, J.; Zeng, L.; Wang, T.; Yi, M.; Song, J. Prenatal diagnosis and pregnancy outcomes in fetuses with vertebral abnormalities. *J. Matern.-Fetal Neonatal Med.* **2025**, *38*, 2468000. https://doi.org/10.1080/14767058.2025.2468000.

124. Cai, X.; Chen, X.; Wei, X.; Liu, W.; Hou, X.; Gong, T.; Zhu, J.; Haacke, E.M.; Wang, G. Use of magnetic resonance imaging in the diagnosis of fetal vertebral abnormalities in utero: a single-center retrospective cohort study. *Quant. Imaging Med. Surg.* **2022**, *12*, 3391–3405. https://doi.org/10.21037/qims-21-1070.

125. Lemire, G.T.; Beauregard-Lacroix, É.; Campeau, P.M.; Parent, S.; Roy-Beaudry, M.; Soglio, D.D.; Grignon, A.; Rypens, F.; Wavrant, S.; Laberge, A.-M.; et al. Retrospective analysis of fetal vertebral defects: Associated anomalies, etiologies, and outcome. *Am. J. Med. Genet. A* **2020**, *182*, 664–672. https://doi.org/10.1002/ajmg.a.61468.

126. Basude, S.; McDermott, L.; Newell, S.; Wreyford, B.; Denbow, M.; Hutchinson, J.; Abdel-Fattah, S. Fetal hemivertebra: associations and perinatal outcome. *Ultrasound Obstet. Gynecol.* **2015**, *45*, 434–438. https://doi.org/10.1002/uog.13401.

127. Zelop, C.M.; Pretorius, D.H.; Benacerraf, B.R. Fetal hemivertebrae: associated anomalies, significance, and outcome. *Obstet. Gynecol.* **1993**, *81*, 412–416.

128. Zhou, H.; Wang, Y.; Huang, R.; Fu, F.; Li, R.; Cheng, K.; Wang, D.; Yu, Q.; Zhang, Y.; Jing, X.; et al. Prenatal Diagnosis and Outcomes in Fetuses with Hemivertebra. *Genes* **2022**, *13*, 1623. https://doi.org/10.3390/genes13091623.

129. Costas, T.; Rodríguez, M.d.l.O.; Martín Esquilas, M.; Alarcón, V.; Goenaga, F.J.; Cabrero, M.Á.; Cubo, A.M. Long-Term Outcomes of Prenatally Diagnosed Fetal Hemivertebra: A 15-Year Single-Center Review. *Children* **2025**, *12*, 1236. https://doi.org/10.3390/children12091236.

130. Horgan, R.; Powel, J.E.; Sham, C.; Spiliopoulos, M.; McLaren, R.A., Jr.; Al-Kouatly, H.B. Genetic etiologies and outcome of isolated fetal hemivertebra: a systematic review. *Am. J. Obstet. Gynecol. MFM* **2023**, *5*, 100813. https://doi.org/10.1016/j.ajogmf.2022.100813.

131. Van den Veyver, I.B.; Chandler, N.; Wilkins-Haug, L.E.; Wapner, R.J.; Chitty, L.S.; ISPD Board of Directors. International Society for Prenatal Diagnosis Updated Position Statement on the use of genome-wide sequencing for prenatal diagnosis. *Prenat. Diagn.* **2022**, *42*, 796–803. https://doi.org/10.1002/pd.6157.

132. Yu, D.; Feng, D.; Qu, J.; Nie, L.; Liu, Q.; Gao, L.; An, W.; Liu, N.; Fang, Y. Application of trio-based whole-exome sequencing in fetal ultrasound anomalies: a single-center retrospective study of 454 cases. *Front. Genet.* **2025**, *16*, 1662801. https://doi.org/10.3389/fgene.2025.1662801.

133. Jiang, M.; Zhang, B.; Wang, J.; Wei, C.; Mao, X.; Yu, B. Exome sequencing and prenatal skeletal abnormalities: comprehensive review and meta-analysis and way forward. *Front. Genet.* **2025**, *16*, 1502538. https://doi.org/10.3389/fgene.2025.1502538.

134. Gao, Z.; Liu, M.; Jiang, J.; Yang, X.; Li, Y.; Zhang, Y.; Wang, Y.; Hua, C.; Liu, N.; Zhu, X.; et al. Whole-genome sequencing for the prenatal evaluation of fetal structural anomalies: a prospective multicenter study. *Am. J. Obstet. Gynecol.* **2026**, *234*, 230–249. https://doi.org/10.1016/j.ajog.2025.08.025.

135. Manickam, K.; McClain, M.R.; Demmer, L.A.; Biswas, S.; Kearney, H.M.; Malinowski, J.; Massingham, L.J.; Miller, D.; Yu, T.W.; Hisama, F.M.; et al. Exome and genome sequencing for pediatric patients with congenital anomalies or intellectual disability: an evidence-based clinical guideline of the American College of Medical Genetics and Genomics (ACMG). *Genet. Med.* **2021**, *23*, 2029–2037. https://doi.org/10.1038/s41436-021-01242-6.

136. Xue, H.; Yu, A.; Zhao, W.; Chen, L.; Fang, R.; Ling, W.; Zhang, L.; Guo, Q.; Lin, N.; Xu, L.; et al. Prenatal diagnosis of fetal skeletal anomalies via whole-exome sequencing in a tertiary referral center. *Sci. Rep.* **2024**, *14*, 27371. https://doi.org/10.1038/s41598-024-75738-x.

137. Yang, L.; Shannon, P.; Silver, R.; Roifman, M.; Yates, C.; Chitayat, D. Prenatal diagnosis of ROR-2 related Robinow syndrome presenting with fetal ultrasound findings of mesomelia, vertebral, digital and genital abnormalities. *Prenat. Diagn.* **2024**, *44*, 653–656. https://doi.org/10.1002/pd.6543.

138. Kelly, M.J.; Alberghina, F.; McCabe, P.; Goldberg, C.J.; Fogarty, E.E.; Dowling, F.E.; O'Toole, P.; Noël, J.; Kiely, P.J.; Moore, D.P.; et al. Functional Outcomes of Congenital Scoliosis at a Mean 35-Year Follow-up Post In Situ Fusion. Revisiting Patients From the 2002 Goldberg et al Study. *J. Pediatr. Orthop.* **2024**, *44*, e381–e388. https://doi.org/10.1097/bpo.0000000000002649.
